# Apelin Prevents and Alleviates Crystalline Silica-induced Pulmonary Fibrosis via Inhibiting Transforming Growth Factor Beta 1-triggered Fibroblast Activation

**DOI:** 10.7150/ijbs.81436

**Published:** 2023-07-31

**Authors:** Jianling Shen, Jiayin Feng, Zhijia Wu, Yushi Ou, Qing Zhang, Qiying Nong, Qifeng Wu, Cong Li, Xiaohui Tan, Meng Ye, Zhongxiang Gao, Ying Zhang, Weihui Liang, Lihua Xia, Yiru Qin, Yongshun Huang, Na Zhao, Shijie Hu

**Affiliations:** 1 Guangdong Provincial Key Laboratory of Occupational Disease Prevention and Treatment, Guangdong Province Hospital for Occupational Disease Prevention and Treatment, Guangzhou, China.; 2 School of Public Health, Sun Yat-Sen University, Guangzhou, China.; 3 School of Public Health, Southern Medical University, Guangzhou, China.; 4 Pudong New Area Center for Disease Control and Prevention, Shanghai, China.; 5 Guangzhou Key Laboratory of Forensic Multi-Omics for Precision Identification, School of Forensic Medicine, Southern Medical University, Guangzhou, China.; 6 National Institute for Occupational Health and Poison Control, Chinese Center for Disease Control and Prevention, Beijing, China.

**Keywords:** apelin, silicosis, pulmonary fibrosis, fibroblast activation, TGF-β1

## Abstract

Silicosis is a common and ultimately fatal occupational disease, yet the limited therapeutic option remains the major clinical challenge. Apelin, an endogenous ligand of the G-protein-coupled receptor (APJ), is abundantly expressed in diverse organs. The apelin-APJ axis helps to control pathological and physiological processes in lung. The role of apelin in the pathological process and its possible therapeutic effects on silicosis have not been elucidated. In this study, we found that lung expression and circulating levels of apelin were markedly decreased in silicosis patients and silica-induced fibrotic mice and associated with the severity. Furthermore, *in vivo* data demonstrated that pre-treatment from day 3 and post-treatment from day 15 with apelin could both alleviate silica-induced pulmonary fibrosis in mice. Besides, apelin inhibited pulmonary fibroblast activation via transforming growth factor beta 1 (TGF-β1) signaling. Our study suggested that apelin could prevent and reverse silica-induced pulmonary fibrosis by inhibiting the fibroblast activation through TGF-β1 signaling pathway, thus providing a new potential therapeutic strategy for silicosis and other pulmonary fibrosis.

## Introduction

Silicosis is a common and ultimately fatal occupational disease, caused by long-term inhalation of crystalline silica. It is a progressive pulmonary disease characterized by extensive chronic inflammation and nodular fibrosis in the lungs 1. Although years of efforts to improve the work environment have been made, the morbidity of silicosis worldwide remains considerable and brings a substantial health burden, especially in developing countries 2. According to the Global Burden of Disease Study 2019 in 204 countries and territories, the number of silicosis new cases increased from 84.4 thousand in 1990 to 139.0 thousand in 2019, with 15.1 thousand to 12.9 thousand deaths in the same period 3. Unfortunately, despite the urgent clinical need, therapies for silicosis are limited, and no effective drugs could be used to reverse its pulmonary fibrosis 4. Although lung transplantation may be the best therapeutic option for late-stage of silicosis patients, it cannot be recommended as a routine treatment because of the limited lung donors and expensive surgery cost 5. Therefore, it is important to investigate the pathological process of silicosis in patients and develop effective therapeutic targets to alleviate the progression of pulmonary fibrosis, thus reducing the mortality of late-stage silicosis.

The occurrence and development of silicosis is a multi-stage complex process involving various cells and factors 6. Growing studies have reported that epithelial cells, macrophages, and fibroblasts participate in the pathogenesis of silicosis 7. After ingestion of silica, alveolar macrophages perpetuate the phagocytosis and cell death processes, bolstering the inflammation and releasing cytokines such as transforming growth factor-β (TGF-β) and tumor necrosis factor-ɑ (TNF-ɑ) 4. Notably, as a key fibrogenic factor, TGF-β-induced fibroblast-to-myofibroblast transition (FMT) is considered the principal event driving the fibrogenic process 8. As a hallmark of silicosis, increased extracellular matrix (ECM) is derived from the dysregulated matrix synthesis by the activation of fibroblasts and the differentiation into myofibroblasts 9. Excessive accumulation of ECM ultimately leads to widespread destruction of lung architecture and reduces pulmonary function 10. Emerging evidence has demonstrated that intervention in the conversion of fibroblasts to myofibroblasts may be a potential strategy for silicosis 9, 11.

Apelin was first described in 1998 as an endogenous ligand of a G protein-coupled receptor (APJ) 12. It is produced from the preproapelin precursor which consists of 77 amino acid residues 13. The apelin/APJ system is expressed in various human tissues and cells 14, 15, particularly high in lung tissues 16, 17. Previous studies showed that apelin was involved in the pathogenesis of several pulmonary diseases, including pulmonary hypertension 18, pulmonary thromboembolism 19, acute lung injury/acute respiratory distress syndrome 20, 21, chronic obstructive pulmonary disease 22, and lung cancer 23. Moreover, accumulating evidence suggests that apelin displays an anti-fibrotic effect in the skin 24, cardiac 25, cardiovascular 26, and renal fibrosis 27, 28, which affects the TGF-β signaling. However, the roles of apelin in the pathological process and the effects on pulmonary fibrosis, including silicosis, and its underlying mechanisms have not been elucidated.

In this study, we investigated the role of apelin in the process of silicosis in patients and mouse models. Moreover, we explored the anti-fibrotic effect and its underlying mechanism of apelin on silicosis both *in vivo* and *in vitro*. Our findings suggest that apelin may be of great potential to treat silicosis and other pulmonary fibrosis.

## Materials and methods

### Clinical samples

In this study, 100 silicosis patients (40 cases of silicosis stage I, 41 cases of stage II, and 19 cases of stage III) and 100 healthy controls were enrolled in Guangdong Province Hospital for Occupational Disease Prevention and Treatment, China. All the collected serum samples were preserved in aliquots at -80 °C. All subjects provided their written informed consent. Human lung tissue samples (paraffin-embedded blocks, healthy controls n=6, stage III silicosis patients n=5) were obtained under the consent of their direct relatives. All healthy control samples and 2 silicosis patient samples were from autopsies of forensic cases at School of Forensic Medicine, Southern Medical University, and 3 silicosis patient samples from National Institute for Occupational Health and Poison Control, Chinese Center for Disease Control and Prevention. This study was conducted after approval by the Medical Ethics Committee of Guangdong Province Hospital for Occupational Disease Prevention and Treatment (No. GDHOD MEC 2010022).

### Pulmonary function tests

Silicosis patients enrolled in this study underwent pulmonary function tests. The pulmonary function parameters including the diffusing capacity of the lungs for carbon monoxide (DLCO), total lung capacity (TLC), forced vital capacity (FVC), forced expiratory volume in one second (FEV1), forced expiratory flow at 50% of the pulmonary volume (FEF50%), and FEF25~75% were measured using a pulmonary function instrument (MIR Spirolab II, MIR Medical International Research, Italy).

### Animal experiments

The animal studies were approved by the Animal Ethics Committee of Guangdong Province Hospital for Occupational Disease Prevention and Treatment (No. GDHOD AEC 2020011). Six to eight-week-old male C57BL/6 mice (18-21 g) were purchased from Guangdong Medical Laboratory Animal Center (Guangzhou, China). All mice were housed under specific pathogen-free conditions and provided food and water ad libitum. Mice were randomly divided into 4 groups as follows: (1) Saline, (2) Saline + Apelin, (3) Silica, and (4) Silica + Apelin. Four groups were instilled intratracheally with 20 μL saline or 250 g/L silica suspension (Sigma, Germany) on day 1, respectively. The Apelin group and Silica + Apelin group were then intraperitoneally injected with 500 μg/kg apelin-13 (Sigma, Germany), which possesses the highest biological activity than other isoforms 29, daily from the 3^rd^ or 15^th^ day after modeling. The lung tissues were collected on the 28^th^ and 56^th^ day after modeling for further analysis.

### Histological analysis

Hematoxylin-eosin (H&E) and Masson trichrome staining were used to evaluate pathological changes in morphology and collagen production in mouse lung tissues, respectively. Immunohistochemical (IHC) staining was used to observe the expression of apelin, APJ, phosphorylated SMAD2/3 (p-SMAD2/3), and F4/80 in lung tissues. Briefly, mouse lung tissues were fixed in 4% paraformaldehyde (Biosharp, Anhui, China) for 48 hours, embedded in paraffin, and sectioned with a thickness of 3 μm. For H&E staining, mouse lung sections were incubated in hematoxylin for 10 mins and eosin for 3 mins. Masson trichrome staining was performed using a Masson trichrome staining kit (MXB Biotech, Fujian, China) according to the manufacturer's protocol. For IHC staining, after antigen retrieval with citrate buffer (pH 6.0) for 3 mins at 210℃ in a pressure cooker, mouse and human paraffin-embedded slices were treated with 3% hydrogen peroxide for 10 mins to block endogenous peroxidase activity, and incubated successively with specific primary antibodies (Table S1) for 1 hour at 37℃, and secondary antibodies for 30 mins at 37℃, and diaminobenzidine for 4 mins at room temperature (RT). The slides were then counterstained with hematoxylin, dehydrated, and coverslipped. Images were acquired using a digital slice scanning system (EasyScan, Motic, China). The degree of pulmonary fibrosis was quantified using modified Ashcroft scoring 30. The IHC scores were evaluated according to the percentage of positive detection and staining intensity. Five fields of each slice were randomly selected and averaged.

### Cell culture

Human fetal lung fibroblast cell line MRC-5 was purchased from KeyGEN Biotechnology Co., Ltd. (KeyGEN, China), and human monocytic cell line THP-1 was purchased from FuHeng Biotechnology Co., Ltd. (FuHeng, China). MRC-5 cells were cultured in MEM (Gibco, USA) supplemented with 10% FBS (Gibco, USA), 1% penicillin/streptomycin (p/s) (Solarbio, USA), 1% nonessential amino acids (Invitrogen, USA), and 1 mM sodium pyruvate (Invitrogen, USA). Human THP-1 cells were cultured in RPMI 1640 (Gibco, USA) supplemented with 10% FBS and 1% p/s. THP1-derived macrophages were obtained by treating THP-1 cells with 100 ng/mL PMA (Sigma, Germany) for 48 hours. All cells were cultured at 37℃ with 5% CO_2_.

### Coculture assay

Approximately 4*10^5^ THP-1 cells were seeded in the upper insert of a six-well Transwell chamber (0.4 µm pore size, Corning, USA) and treated with 100 ng/mL PMA for 48 hours to differentiate into THP1-derived macrophages. After that, the inserts were washed with PBS to remove PMA and placed into another six-well plate seeded with MRC-5 cells (2*10^5^/well) in advance. After treatment with SiO_2_ or the TGF-β1 inhibitor SB431542 (Selleck, USA) for 48 hours, cell supernatants and cell lysates were collected for subsequent assays.

### Cell viability assay

To assess the viability of MRC-5 cells or THP-1-derived macrophages treated with SB431542, apelin, or SiO_2_, respectively. A Cell Counting Kit-8 (CCK8) assay (Dojindo Laboratories, Japan) was used. Cells in 100 μL medium were seeded at a density of 3*10^3^/well (MRC-5 cells) or 1*10^4^/well (THP-1 cells) in 96-well plates for 24 or 48 hours. After being treated with different concentrations of targeted reagents for 48 hours, cells in each well were incubated with 10 μL CCK-8 solution at 37℃ for 2 hours. The absorbance was measured at 450 nm with a microplate reader (Multiskan GO, Thermo Scientific, USA). Cell viability was calculated as relative viability with reference to untreated control (100%).

### Immunofluorescence (IF) analysis

Lung tissue sections and cell crawling slides were fixed in 4% paraformaldehyde. Paraffin-embedded lung tissue sections were deparaffinized and antigen repaired with citrate buffer (pH 6.0) for 3.5 mins at 210℃ in a pressure cooker. Afterward, slides were treated with 3% hydrogen peroxide for 10 mins to block endogenous peroxidase activity, permeabilized by 0.1% Triton X-100 for 15 mins, and blocked with 5% bovine serum albumin for 2 hours at RT. Then, slides were incubated with the selected primary antibodies overnight at 4°C: (i) APJ co-stained with alpha-smooth muscle actin (α-SMA) for the expression of APJ on activated fibroblasts. (ii) α-SMA for the activation of fibroblasts in lung tissues; (iii) APJ for the expression of APJ in MRC-5 cell line; (iv) α-SMA for the activation of MRC-5 cells. Next, the samples were incubated with appropriate fluorescent secondary antibodies for 1 hour at RT (Table S1). The nuclear was stained with 4',6-Diamidino-2-phenylindole (DAPI). Stained slides were observed under a confocal microscope (LSM 980, Zeiss, Germany) or fluorescence microscope (BX61, Olympus, Japan).

### Scratch assay

Cell migratory capacity was assessed by scratch assay. 2*10^5^ MRC-5 cells were seeded in a six-well plate with a marked line at the bottom, and cultured in an FBS-free MEM medium for 24 hours. Next, wounds were made using 200 µl pipette tips. After washing with PBS, FBS-free MEM medium containing selected drugs was added to the scratched MRC-5 cells. Optical images were captured at 0, 24, and 48 hours using a microscope (IX71, Olympus, Japan). The migration rate was quantified by ImageJ software.

### Enzyme-linked immunosorbent assay (ELISA)

The levels of apelin in the serum of healthy controls and silicosis patients or mice were detected by Human Apelin ELISA kits or Mouse Apelin ELISA kits (Novus Biologicals, USA), respectively. This ELISA kit has cross-reactivity with apelin isoforms, therefore, the summation of serum apelin-13, apelin-17, apelin-28, apelin-31, and apelin-36 was measured. The levels of TGF-β1 in supernatants of THP-1 macrophages were measured with a TGF-β1 ELISA kit (Thermo Scientific, USA). All procedures were performed according to the manufacturer's protocol. Briefly, samples were added into pre-coated ELISA plates and incubated with corresponding detection antibody and HRP-conjugate, successively. After removing unbound proteins with washing buffer, the samples were incubated with substrate solution, and then the enzyme reaction was stopped by stop solution. The absorbance was measured at 450 nm with a microplate reader (Multiskan GO, USA).

### Real-time quantitative PCR (RT-qPCR)

Total RNA was extracted from lung tissues or MRC-5 cells using TRIzol^®^ reagent (Invitrogen, USA). RNA concentration and purity were assessed by the Nanodrop 2000 spectrophotometer (Thermo Scientific, USA). After that, total RNA was reversely transcripted to cDNA by the One-Step reverse transcription kit (TransGen Biotech, China) in a thermal cycler (Analytik Jena AG, Germany). qPCR was progressed by LightCycler® 96 (Roche, Germany) real-time PCR instrument using PerfectStart Green qPCR SuperMix (TransGen Biotech, China). Relative expression levels of targeted genes were calculated using the 2^-ΔΔ Ct^ method. *β-actin* mRNA was tested as the internal control. All primer sequences are presented in Table S2.

### Western blotting assay

Total protein was extracted from lung tissues or MRC-5 cells using RIPA lysis buffer (KeyGEN, China) containing proteinase and phosphatase inhibitor. The protein concentration was measured with the BCA protein assay kit (Solarbio, USA). Aliquots of 20-40 µg proteins were separated with 8%, 12%, or 15% SDS-polyacrylamide gel electrophoresis (SDS-PAGE) and transferred to PVDF membranes (Millipore, USA). After blocking with 5% skim milk (Biofroxx, Germany) or bovine albumin (Solarbio, USA), membranes were incubated with specific primary antibodies overnight at 4℃, then incubated with secondary antibodies at RT for 1.5 hours. Finally, an enhanced chemiluminescence detection kit (KeyGEN, China) was used to detect proteins and the ImageJ software was used for the grayscale analysis of protein bands. The antibodies used for western blotting are also listed in Table S1.

### Plasmid and transient transfection

The human SMAD family member 2 (*SMAD2*) (RefSeq NM_005901.6) cDNA was cloned into pcDNA3.1-GFP which encodes the green fluorescent protein (GFP), while the human SMAD family member 3 (*SMAD3*) (RefSeq NM_005902.4) cDNA was cloned into pcDNA3.1-mCherry which encodes the red fluorescent protein. These plasmids were synthesized by Tsingke Biotechnology Ltd (Tsingke, China). Amplification of plasmid DNA was performed using the *Escherichia coli* strain. Plasmid DNA was purified using the QIAGEN Plasmid Midi Kit (QIAGEN, Germany).

Plasmid transfection was carried out using Lipofectamine 3000 transfection reagent (Invitrogen, USA). Briefly, MRC-5 cells were seeded in a 6-well plate to be at 70% confluence on the day of transfection and were transfected using the transfection reagent. 24 hours later, GFP and mCherry fluorescence were observed for a rough assessment of transfection efficiency, and Western blotting was performed for SMAD2 and SMAD3 overexpression confirmation. After transfection for 24 hours, the medium was changed and MRC-5 cells were further stimulated with TGF-β1 (10 ng/mL) or apelin (100 nM) for 48 hours. Cells were harvested for extraction of proteins and RNA for further detection.

### Statistical analysis

Statistical analyses were carried out by SPSS 26.0 and GraphPad Prism 8.0.2 software. Data were presented as means ± standard error of mean (SEM). Student's t-test was used for two-group comparisons and one-way analysis of variance (ANOVA) was used for multiple-group comparisons. The correlation of various pulmonary function parameters and serum apelin levels in silicosis patients was estimated by partial correlation analysis after adjustment for age and smoking. A Spearman correlation coefficient <0.30 was considered to be irrelevant. *P* value <0.05 was considered statistically significant.

## Results

### Apelin levels decrease dramatically and positively correlate with pulmonary functions in silicosis patients

**Figure 1:**
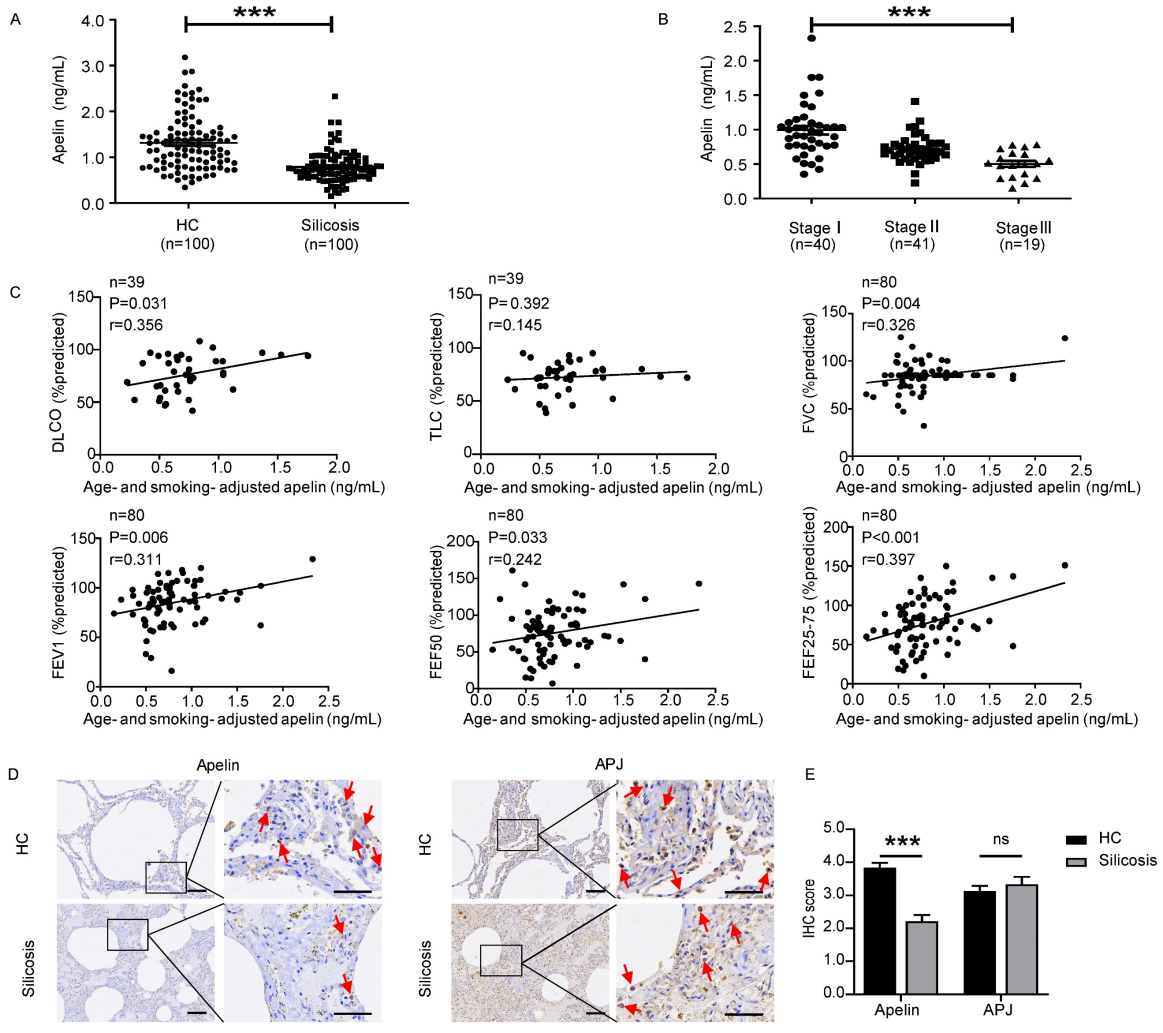
** Apelin levels decrease dramatically and positively correlate with pulmonary functions in silicosis patients. (A)** Serum levels of apelin in healthy controls (n=100) and silicosis patients (n=100). **(B)** Serum levels of apelin in silicosis patients with stage I (n=40), stage II (n=41), and stage III (n=19).** (C)** Partial correlation of lung function (DLCO, TLC, FVC, FEV1, FEF50, and FEF25-75% predicted) and age- and smoking- adjusted serum apelin levels. **(D)** Representative images of apelin and APJ immunostaining in lung tissues from healthy controls (n=6) and silicosis patients (n=5). The boxed regions are shown at higher magnification in the right panels. The red arrows show positive cells. Scale bar: 25 µm. **(E)** The immunohistochemical scores of apelin and APJ. Data are presented as means ± SEM for at least triplicate experiments. *P* > 0.05 is considered not significant (ns), and ***P < 0.001.

In recent years, apelin has been found to be associated with several lung diseases 18-22. However, the role of apelin in pulmonary fibrosis has not yet been revealed. Therefore, we first investigated serum apelin levels of silicosis patients by ELISA. Interestingly, we found that serum apelin levels decreased considerably in silicosis patients compared with controls (*P*<0.05) (Figure **1A**). Meanwhile, serum apelin levels were significantly lower in stage III patients compared to stage II (*P*<0.05) as well as stage II compared to stage I (*P*<0.05) (Figure **1B**). More strikingly, after adjusting for age and smoking, serum apelin levels were positively correlated to DL_CO_% (*P*=0.031), FVC% (*P*=0.004), FEV1% (*P*=0.006), FEF50% (*P*=0.033), and FEF25-75% (*P*<0.001) in silicosis patients (Figure **1C**). These data suggest that apelin levels are declined in patients who suffer from more severe forms of silicosis with impaired pulmonary function.

Since apelin is widely distributed in various organs 14, 15, serum apelin levels may not accurately represent the apelin levels in lung tissue. Therefore, we further performed IHC staining for apelin and APJ in lung sections from five silicosis patients and six healthy controls. Apelin and APJ protein were observed in alveolar epithelial cells, bronchial epithelial cells, and fibroblasts (Figure **1D**). More importantly, IF co-staining of APJ and α-SMA (a specific marker of myofibroblasts) showed that APJ protein was observed in activated fibroblasts in silicosis patients (Figure **S1A**). Compared to healthy controls, apelin protein expressions were decreased in silicosis lung samples (*P*<0.05) while APJ protein expressions remained unchanged (*P*>0.05) (Figure **1E**). Taken together, these results indicate that apelin is decreased in silicosis patients and positively correlated to pulmonary functions.

### Pulmonary apelin expression reduces dramatically in silicotic mice

**Figure 2:**
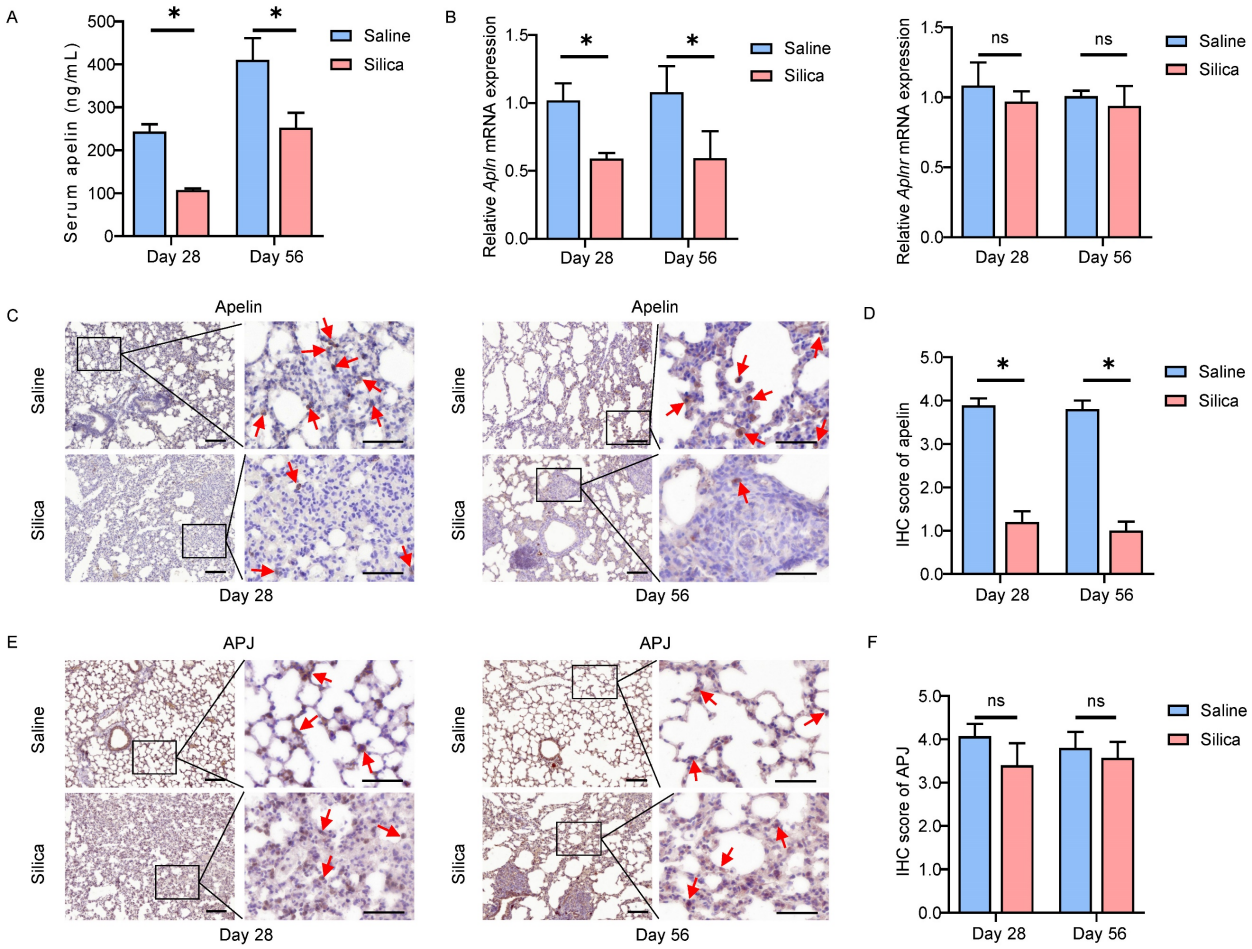
** Apelin expression reduces dramatically in silicotic mice. (A)** Serum levels of apelin in saline- and silica- treated mice (n=5). **(B)** The mRNA expression of *Apln* and *Aplnr* in saline- and silica- treated mice lungs (n=5). **(C** and **E)** Representative images of apelin and APJ immunostaining in lung tissues from saline- and silica- treated mice (n=5). The boxed regions are shown at higher magnification in the right panels. The red arrows show positive cells. Scale bar: 25 µm. **(D** and **F)** The immunohistochemical scores of apelin and APJ. Data are presented as means ± SEM for at least triplicate experiments. *P* > 0.05 is considered not significant (ns), *P < 0.05.

Similar observations were confirmed in *in vivo* silica-induced mouse models as well. After 28 or 56 days of silica administration, the serum apelin levels were significantly lower than that of the saline-treated group (*P*<0.05) (Figure **2A**). In addition, the *Apln* (mouse gene for apelin) mRNA levels in lung tissues also reduced in the silicotic mice (*P*<0.05), while *Aplnr* (mouse gene for APJ) mRNA levels remained unchanged (*P*>0.05) (Figure **2B**). Likewise, apelin and APJ IHC staining in lung tissues from saline- and silica- challenged mice showed that both apelin and APJ protein were observed in alveolar epithelial cells, bronchial epithelial cells, and fibroblasts (Figure** 2C, E**). Similarly, APJ protein was also observed in activated fibroblasts in lung tissues of silicotic mice by IF staining (Figure **S1B**). Compared to saline controls, apelin protein was lower in silicotic lung samples (*P*<0.05) (Figure **2D**) while APJ protein remained stable (*P*>0.05) (Figure **2F**). These data in mouse models further indicate that apelin expression is decreased in silicosis. As a membrane protein receptor, APJ expression is relatively stable.

### Apelin prevents silica-induced pulmonary fibrosis

**Figure 3:**
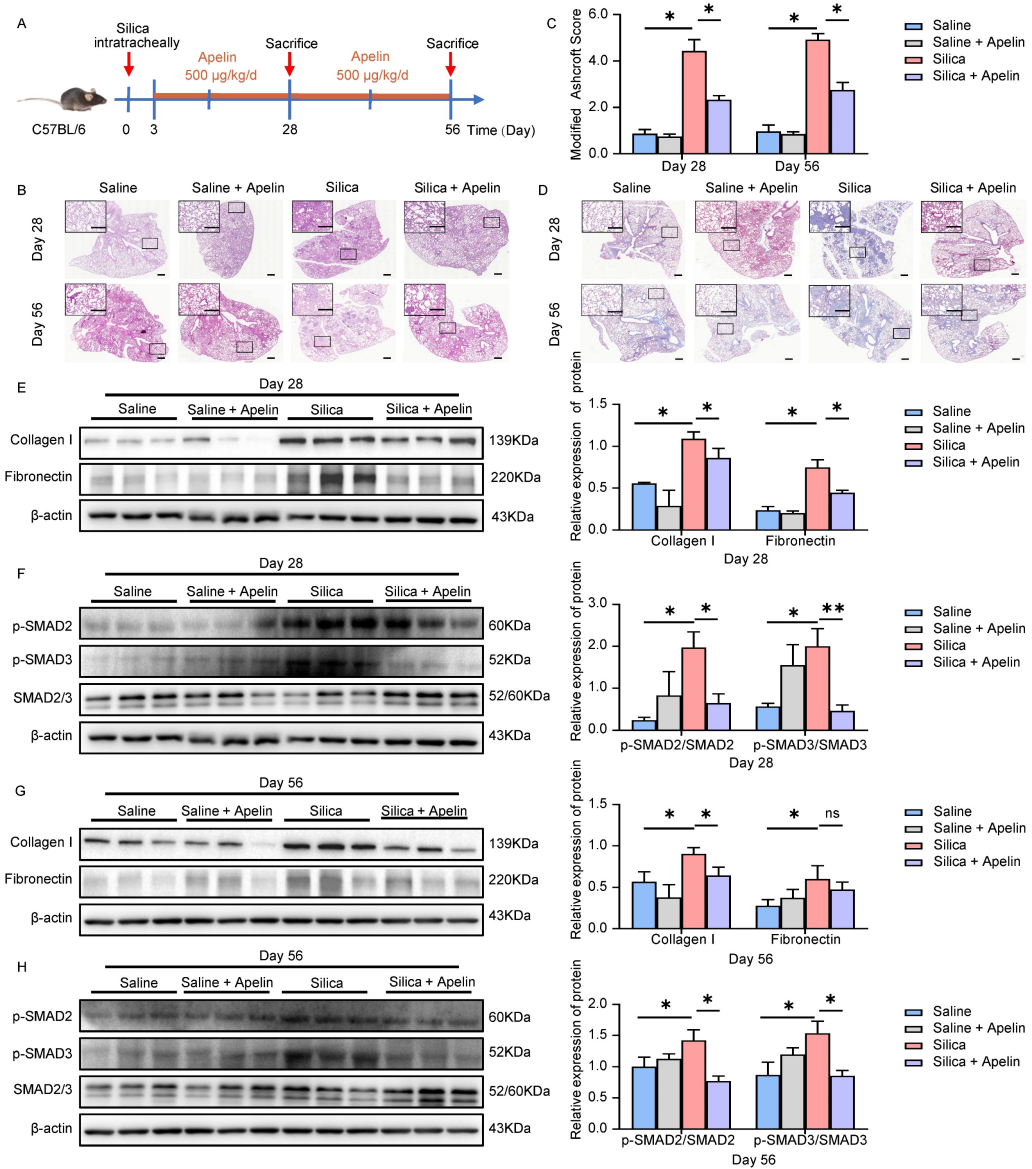
** Apelin prevents silica-induced pulmonary fibrosis. (A)** Schematic diagram of early intervention with apelin in the silica- induced mouse model. Apelin (500 μg/kg) was intraperitoneally injected from day 3 after silica administration, and lungs were assessed on days 28 and 56. **(B)** Representative H&E staining of lung sections from saline- or apelin- treated mice. The boxed regions are shown at higher magnification in the right panels. Scale bar: 100 µm. **(C)** Modified Ashcroft score in the lungs of saline- or apelin- treated mice (n=5). **(D)** Representative Masson's trichrome staining of lung sections from saline- or apelin- treated mice. The boxed regions are shown at higher magnification in the right panels. Scale bar: 100 µm. **(E** and** G)** Western blotting analysis of collagen I and fibronectin in lung homogenates of saline- or apelin- treated mice and their quantification (n=3). β-actin was used as a loading control. **(F** and** H)** Western blotting analysis of phosphorylation and total expression of SMAD2 and SMAD3 in lung homogenates of saline- or apelin- treated mice and their quantification (n=3). β-actin was used as a loading control. Data are presented as means ± SEM for at least triplicate experiments. *P* >0.05 is considered not significant (ns), *P < 0.05, and **P < 0.01.

Given that pulmonary expression and circulating levels of apelin were diminished in both silicosis patients and silicotic mice, the lack of apelin may impair pulmonary function. Therefore, we next applied exogenous apelin to silicotic mouse models to explore its effects against silica-induced pulmonary fibrosis. Firstly, we investigated the preventive potential of apelin against pulmonary fibrosis. Apelin was intraperitoneally administrated daily at 500 μg/kg from day 3 after silica exposure. Lung tissues were collected for further analysis on both the 28^th^ and 56^th^ day (Figure **3A**). We first assessed the level of pulmonary fibrosis between silicosis and apelin-treated lungs using several tissue staining methods. H&E staining of lung sections revealed less degree of alveolar septal thickening and silicon nodules in apelin-treated lungs than in silicosis lungs (Figure **3B**). Additionally, a lower Ashcroft score in apelin-treated lung sections than in silicosis lung sections was observed (*P*<0.05) (Figure **3C**). Masson's trichrome staining indicated more collagen deposition in the silicosis group than apelin-treated group (Figure **3D**). Western blotting analysis of collagen type I (collagen I) and fibronectin in lung lysates were reduced in the apelin-treated group as compared to the silicosis group (Figure **3E, G**). These data show that early intervention with apelin can effectively prevent the progression of silica-induced pulmonary fibrosis in mice.

The canonical TGF-β1/SMADs signaling pathway is considered of great importance in pulmonary fibrosis 8. This signaling pathway involves the phosphorylation and activation of SMAD2 and SMAD3 by TGF-β receptor 1 (TGFR1) 31. The levels of p-SMAD2/3 are used as the indicators of TGF-β signaling activity 32-36. Notably, we observed that the number of p-SMAD2/3-positive cells was significantly elevated in both silicosis patients (Figure **S2A-B**) and silicotic mice lung tissues (Figure **S2C-D**). We then evaluated the effect of apelin on the TGF-β1/SMADs signaling. Protein expressions of lung lysates from silica- and/or apelin- treated mice were assessed using western blotting assay. Our results showed that silica exposure increased the phosphorylation of SMAD2 and SMAD3 (Figure **3F, H**). On the contrary, after apelin intervention, the protein levels of p-SMAD2 and p-SMAD3 were inhibited (Figure **3F, H**). These data suggest that apelin prevents the progression of silica-induced pulmonary fibrosis probably by blocking the TGF-β1/SMADs signaling pathway.

### Apelin reverses silica-induced pulmonary fibrosis

**Figure 4:**
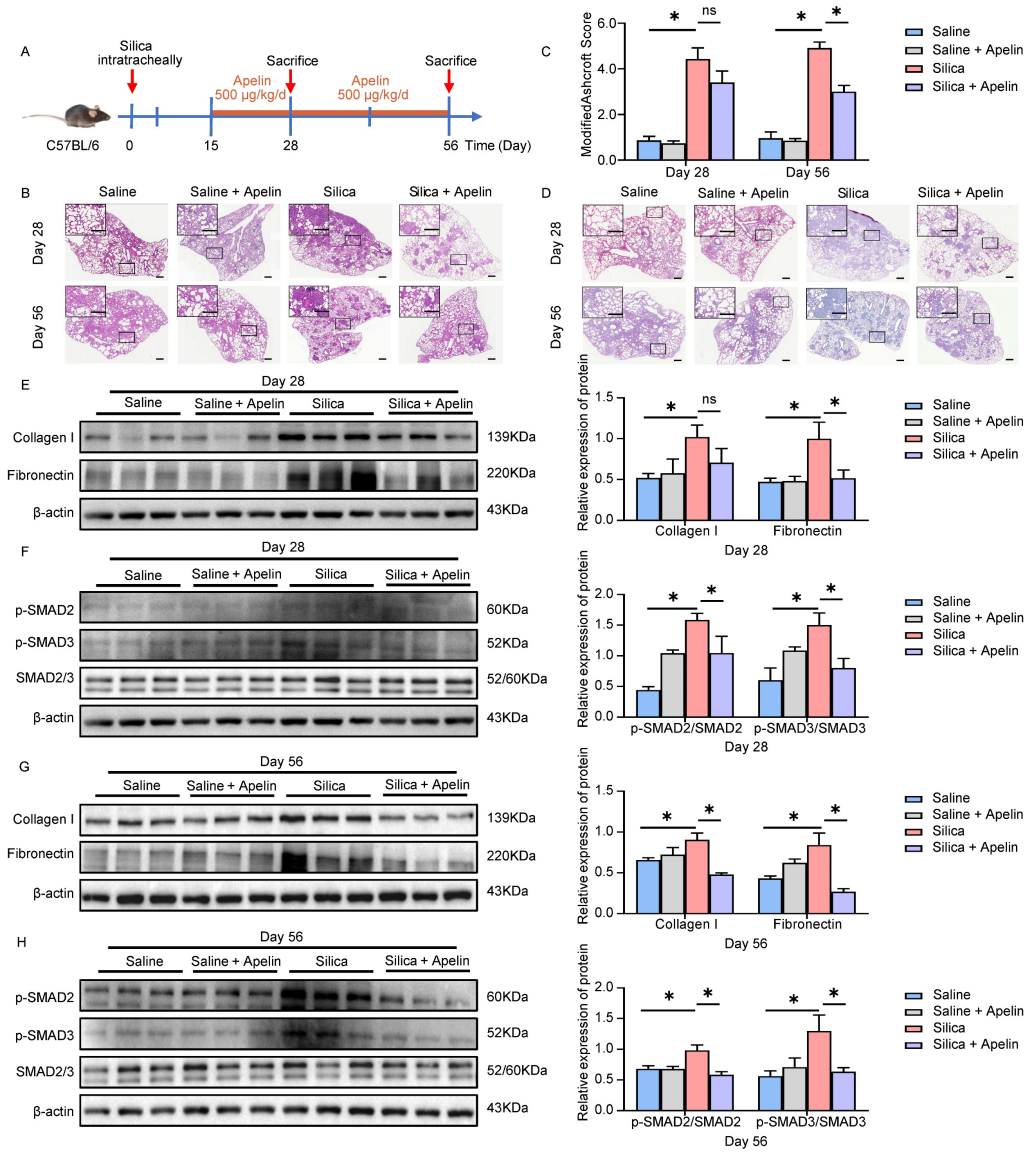
** Apelin reverses silica-induced pulmonary fibrosis. (A)** Schematic diagram of treatment with apelin in the therapeutic lung fibrosis model. Mice were injected intraperitoneally with apelin (500 μg/kg) starting on day 15, and lungs were assessed on days 28 and 56 after silica administration. **(B)** Representative H&E staining of lung sections from saline- or apelin- treated mice. The boxed regions are shown at higher magnification in the right panels. Scale bars, 100 µm.** (C)** Fibrosis score in the lungs of saline- or apelin- treated mice (n=5). **(D)** Representative Masson's trichrome staining of lung sections from saline- or apelin- treated mice. The boxed regions are shown at higher magnification in the right panels. Scale bar: 100 µm. **(E** and** G)** Western blotting analysis of collagen I and fibronectin in lung homogenates of saline- or apelin- treated mice and their quantification (n=3). β-actin was used as a loading control. **(F** and** H)** Western blotting analysis of phosphorylation and total expression of SMAD2 and SMAD3 in lung homogenates of saline- or apelin- treated mice and their quantification (n=3). β-actin was used as a loading control. Data are presented as means ± SEM for at least triplicate experiments. *P* > 0.05 is considered not significant (ns), *P < 0.05.

Numerous silicosis patients are diagnosed after obvious clinical symptoms and imaging appearing 37. Therefore, we further determined whether apelin could be effective against silica-induced pulmonary fibrosis when treating mice later in the disease course (administrated from day 15 to 28 or 56 after modeling) (Figure **4A**). The results showed that late treatment with apelin alleviated the fibrous nodules and destruction of alveolar structures (Figure **4B**) and significantly reduced Ashcroft scores (*P*<0.05) (Figure **4C**). The late treatment of apelin also alleviated collagen deposition (Figure **4D**) and reduced the protein levels of collagen I and fibronectin in the lungs on days 28 and 56 after silica administrating (Figure **4E, G**). These data indicate that late treatment with apelin promotes the resolution of pulmonary fibrosis in silica-induced mice. Similar to early intervention, the late treatment also reduced the phosphorylation of SMAD2 and SMAD3 (Figure **4F, H**).

### TGF-β1 stimulation decreases apelin synthesis in fibroblasts

**Figure 5:**
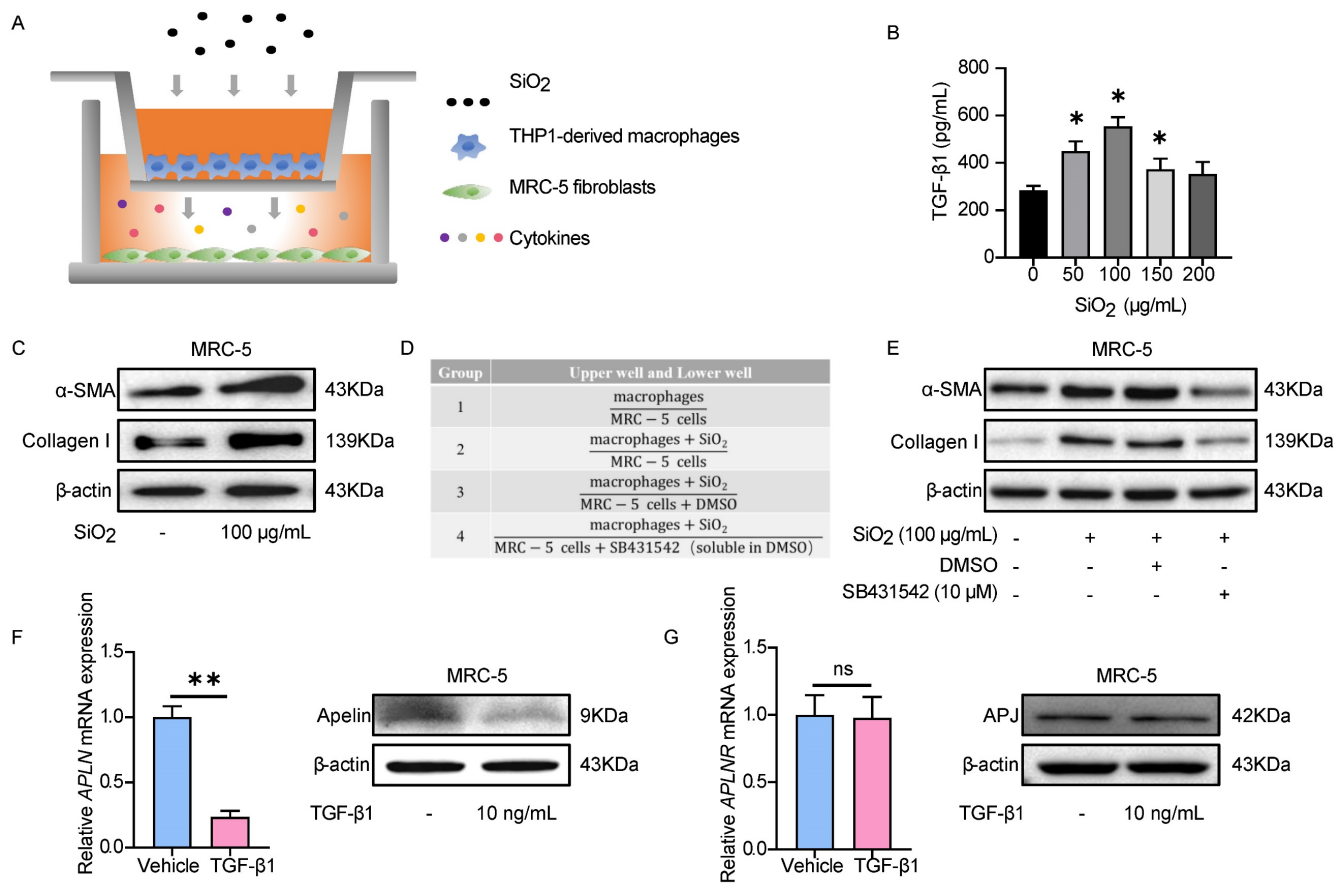
** TGF-β1 stimulation decreases apelin synthesis in fibroblasts. (A)** Schematic model of the co-culture system of THP1-derived macrophages and MRC-5 fibroblasts. MRC-5 fibroblasts were co-cultured for 48 hours with THP1-derived macrophages that had been pretreated with or without SiO_2_. **(B)** The levels of TGF-β1 in the supernatant of THP1-derived macrophages after treatment with different doses of SiO_2_ for 48 hours. **(C)** The protein expression of α-SMA and collagen I in MRC-5 cells in the co-culture system. β-actin was used as a loading control. **(D)** Four grouping schematics of the co-culture system. MRC-5 fibroblasts were co-cultured for 48 hours with THP1-derived macrophages that had been pretreated with or without SiO_2_, and SB431542 or DMSO were added to some co-culture groups. **(E)** Western blotting analysis of α-SMA and collagen I expression in MRC-5 cells in the co-culture system. β-actin was used as a loading control. **(F)** Apelin protein and *APLN* mRNA expression in MRC-5 cells after treatment with or without TGF-β1 for 48 hours. β-actin was used as a loading control. **(G)** APJ protein and *APLNR* mRNA expression in MRC-5 cells after treatment with or without TGF-β1 for 48 hours. β-actin was used as a loading control. Data are presented as means ± SEM for at least triplicate experiments. *P* > 0.05 is considered not significant (ns), *P < 0.05, and **P < 0.01.

The activation of fibroblasts and transdifferentiation into myofibroblasts is one of the most important cytopathological events in pulmonary fibrosis 38. During silica-induced pulmonary fibrosis, pulmonary alveoli macrophages are activated by inhaled crystalline silica, release a variety of cytokines and promote fibroblasts to proliferate into α-SMA-positive myofibroblasts, resulting in subsequent excessive deposition of ECM and destruction of the lung architecture 39. We also observed silica-induced macrophage infiltration and fibroblast activation in both silicosis patients and silicotic mice lung tissues by staining of F4/80 and α-SMA, respectively (Figure **S3A-D** and** S4A-B**). Thus, an *in vitro* co-culture model of THP1-derived macrophages and MRC-5 fibroblasts was established to simulate this complex process of fibrosis (Figure **5A**). Since TGF-β1 is the main cytokine factor promoting FMT process secreted by macrophages 40, we measured the levels of TGF-β1 in the upper wells culture medium of co-culture model by ELISA. The level of TGF-β1 reached its maximum concentration at the SiO_2_ concentration of 100 μg/mL (Figure **5B**), and α-SMA and collagen I protein elevated in MRC-5 cells (Figure **5C**), indicating that MRC-5 fibroblasts could be activated in this co-culture model. To determine whether SiO_2_-treated macrophages activated MRC-5 cells depend on TGF-β1, we used TGF-β1 receptor inhibitor SB431542 to completely abrogate any effects by TGF-β1 (Figure **5D**). As expected, SB431542 inversed the increase of α-SMA and collagen I expression in MRC-5 cells co-cultured with SiO_2_-treated macrophages (Figure **5E**), and no significant cell toxicity was observed (Figure** S5**).

Given that we have confirmed that SiO_2_-treated macrophages activated MRC-5 cells depending on TGF-β1, commercialized TGF-β1 was used for subsequent experiments. Based on our preliminary results for TGF-β1-induced α-SMA expression in MRC-5 fibroblasts (Figure **S6A-B**), treatment with 10 ng/mL TGF-β1 for 48 hours was chosen for the subsequent experiments. First, we detected the expression of apelin in fibroblasts after TGF-β1 stimulation. *APLN* (human gene for apelin) mRNA and protein expressions were significantly inhibited by TGF-β1 treatment in MRC-5 cells (Figure **5F**), consistent with our findings in silicosis patients and silicotic mice. In contrast, *APLNR* (human gene for APJ) mRNA and protein expression were not changed after TGF-β1 stimulation (Figure **5G**). These data indicate that TGF-β1 secreted by SiO_2_-treated macrophages induces the activation of MRC-5 fibroblasts, and the synthesis of apelin is decreased in this process.

### Apelin suppresses the activation, migration, and ECM synthesis of fibroblasts triggered by TGF-β1

**Figure 6:**
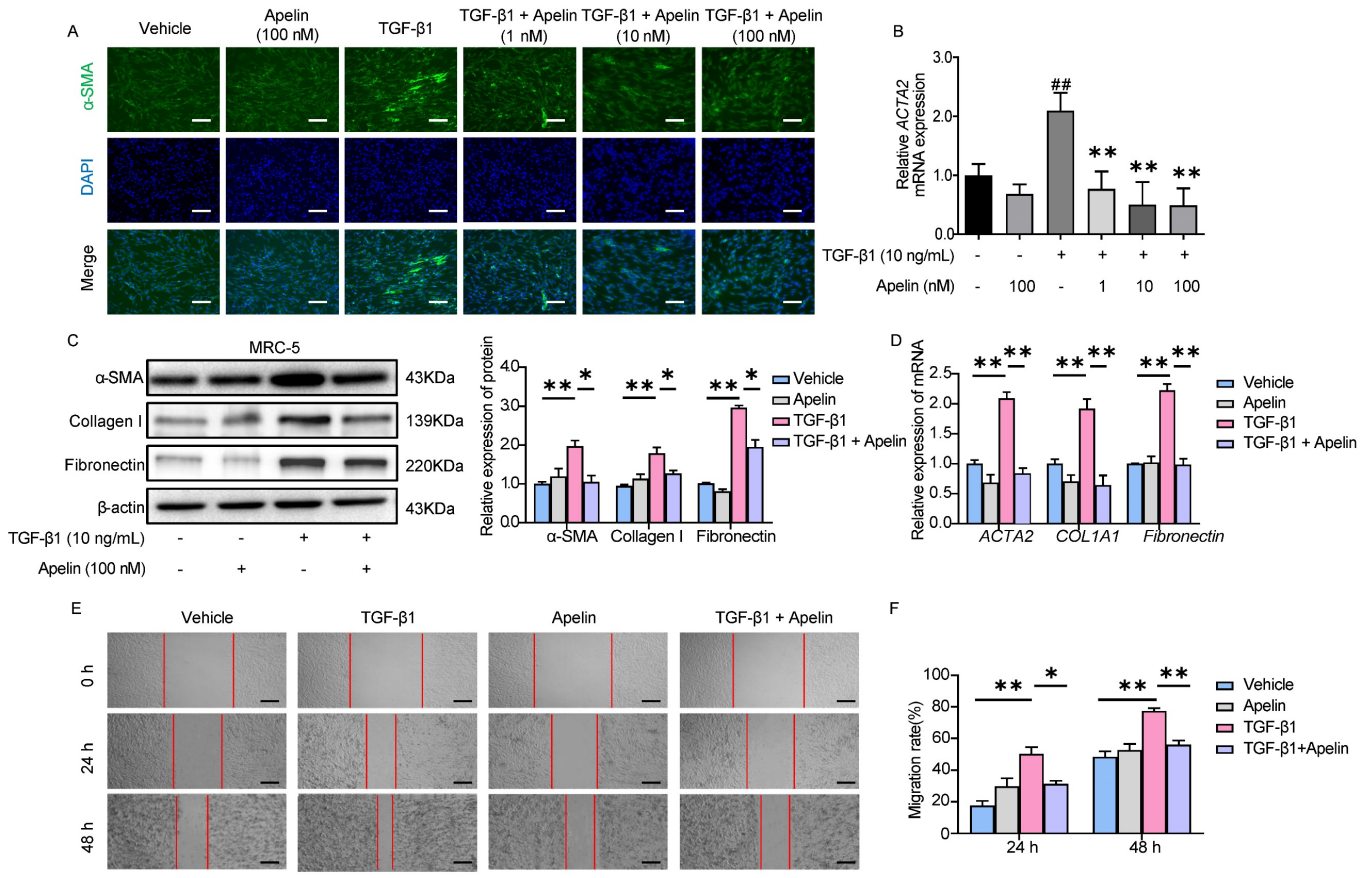
** Apelin suppresses the activation, migration, and ECM synthesis of fibroblasts triggered by TGF-β1.** MRC-5 fibroblasts were treated with apelin (100 nM) or vehicle control in the absence or presence of TGF-β1 (10 ng/mL) for 48 hours. **(A)** Representative images of α-SMA immunostaining in MRC-5 cells. Cells were counterstained with DAPI to visualize nuclei. Scale bar: 100 µm. **(B)** The mRNA expression of *ACTA2* in MRC-5 cells. ^##^*P* < 0.01 *vs.* vehicle group; **P < 0.05 *vs.* TGF-β1 group. **(C)** Western blotting analysis of α-SMA, fibronectin, and collagen I expression in MRC-5 cells and their quantification. β-actin was used as a loading control. **(D)** The mRNA expression of *ACTA2*, *COL1A1*, and *Fibronectin* in MRC-5 cells. **(E)** Images of scratch in MRC-5 cells. Scale bar: 100 µm. **(F)** The migration rate of MRC-5 cells calculated from the scratch assay. Data are presented as means ± SEM for at least triplicate experiments. *P* > 0.05 is considered not significant, *P < 0.05, and **P < 0.01.

To explore the inhibitory effect of apelin on fibroblasts, we assessed the effects of adding apelin to MRC-5 cells. APJ expression on the surface of MRC-5 cells was confirmed by cellular IF (Figure **S7**). α-SMA protein and *ACTA2* (gene for α-SMA) mRNA overexpressions were inhibited by the addition of apelin in a dose-dependent manner as shown by immunofluorescence staining and RT-PCR (Figure **6A-B**), and no significant cell toxicity was observed (Figure **S8A-B**). TGF-β1-induced α-SMA, relative ECM synthesis proteins collagen I and fibronectin expressions were also alleviated by adding apelin at the high concentration (100 nM) (Figure **6C-D**). Additionally, apelin inhibited MRC-5 cell migration induced by TGF-β1 using scratch assay (Figure **6E-F**). To sum up, these results suggest that apelin suppresses TGF-β1-induced activation, migration, and ECM production of MRC-5 fibroblasts.

### Apelin inhibits TGF-β-SMAD2/3 signaling and downregulates the expression of *SNAI1* and *SNAI2*

**Figure 7:**
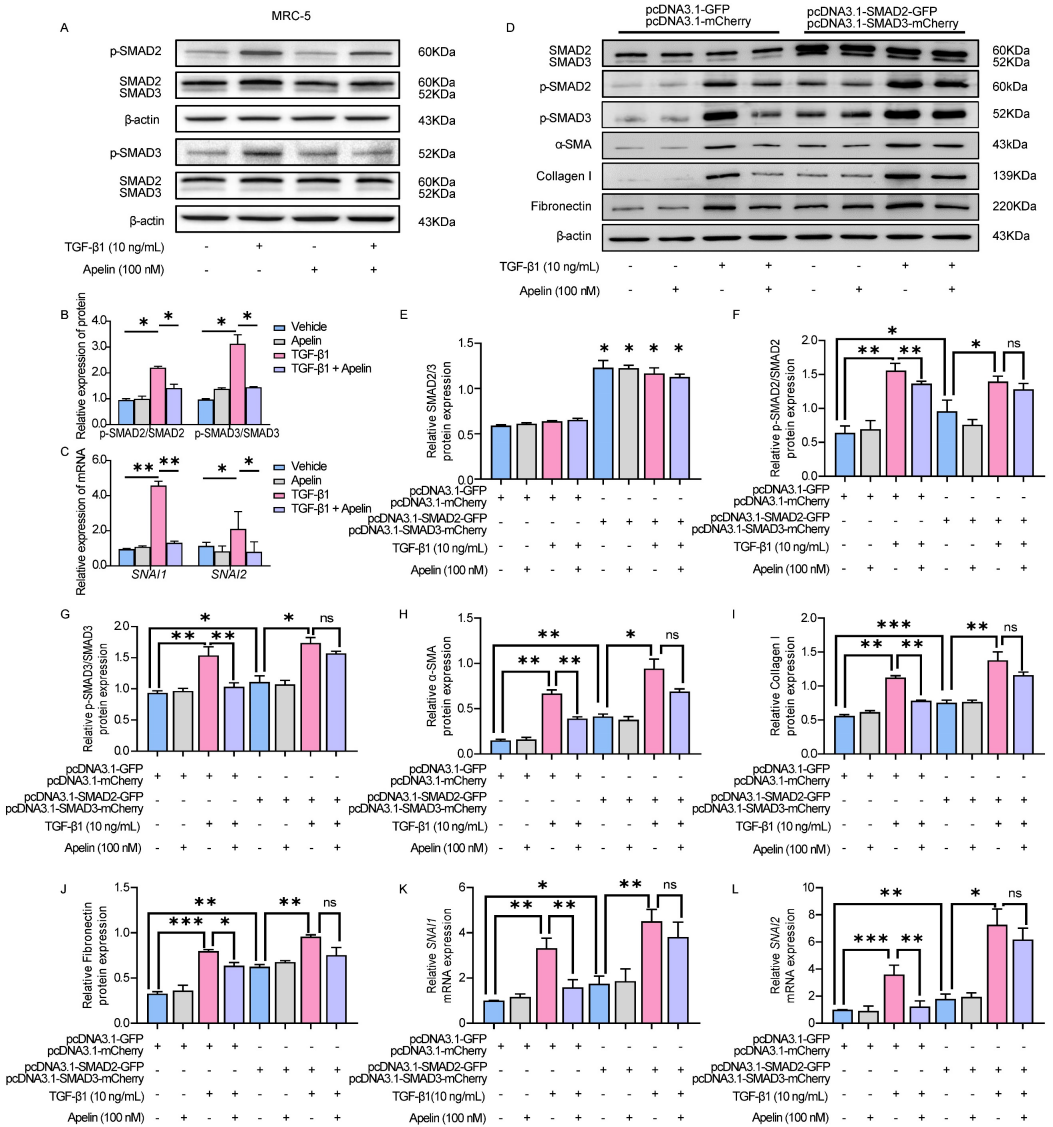
** Apelin inhibits TGF-β-SMAD2/3 signaling and downregulates the expression of *SNAI1* and *SNAI2*. (A-C)** MRC-5 fibroblasts were treated with apelin (100 nM) or vehicle control in the absence or presence of TGF-β1 (10 ng/mL) for 48 hours. **(A** and** B)** Western blotting analysis of the phosphorylated and total expression of SMAD2 and SMAD3 in cell lysates of MRC-5 cells and their quantification. β-actin was used as a loading control.** (C)** The mRNA expression of *SNAI1* and *SNAI2* in MRC-5 cells. **(D-L)** MRC-5 fibroblasts were co-transfected with pcDNA3.1-SMAD2-GFP and pcDNA3.1-SMAD3-mCherry plasmid or pcDNA3.1-GFP and pcDNA3.1-mCherry plasmid for 24 hours, then treated with apelin (100 nM) in the absence or presence of TGF-β1 (10 ng/mL) for 48 hours. **(D-J)** Western blotting analysis of SMAD2/3, p-SMAD2, p-SMAD3, α-SMA, collagen I, and fibronectin expression in MRC-5 cells and their quantification. β-actin was used as a loading control.** (K** and** L)** The mRNA expression of *SNAI1* and *SNAI2* in MRC-5 cells. Data are presented as means ± SEM for at least triplicate experiments. *P* > 0.05 is considered not significant (ns), *P < 0.05, **P < 0.01, and ***P < 0.001.

It has been known that TGF-β1 induces α-SMA, collagen I, and fibronectin production via p-SMAD2/3 41. To better understand the molecular mechanism of the inhibitory effects of apelin in silicosis, we examined the possible involvement of the key TGF-β1/SMAD pathway. The results showed that there was no significant change in total SMAD2/3, but the level of p-SMAD2/3 was notably increased after TGF-β1 stimulation. Inversely, apelin treatment significantly decreased the phosphorylation of SMAD2/3 (Figure **7A-B**). Moreover, the expressions of transcription factors snail family transcriptional repressor 1 (*SNAI1*) and snail family transcriptional repressor 2 (*SNAI2*, previously known as *SLUG*), which are key regulators mediated TGF-β1/SMAD signaling triggered the ECM proteins overexpression and deposition in pulmonary fibrosis, were downregulated by the treatment of apelin (Figure **7C**).

To examine whether SMAD2/3 contributes to apelin mediated protective effects *in vitro*, we overexpressed SMAD2/3 in MRC-5 by plasmid transfection. The green and red fluorescence indicated the transfection efficiency of control plasmids or SMAD2/3 plasmids were both about 50% ~70% in MRC-5 (Figure **S9**). We observed that SMAD2/3 overexpression alone induced upregulation of fibrosis markers (collagen I, fibronectin, and α-SMA) and transcription factors *SNAI1* and *SNAI2*, and this pro-fibrotic effect was further aggravated by TGF-β1 treatment (Figure **7D-L**). The fibrosis-inhibitory effect of apelin was diminished by SMAD2/3 overexpression (Figure **7D-J**), as well as the insignificant down-regulation of *SNAI1* and *SNAI2* expression (Figure **7K-L**). The data imply that apelin has inhibitory effects on the activation of TGF-β-SMAD2/3 signaling, and downregulates TGF-β triggered *SNAI1* and *SNAI2* expressions in fibroblasts.

## Discussion

Apelin, a novel peptide identified as the endogenous ligand to the APJ, has been reported reduced expression associated with renal, skin, myocardial, and pulmonary artery fibrosis 18, 24, 42, 43. Consistent with these studies, we demonstrated that apelin expression was significantly decreased in serum and lung in both silicosis patients and mice. Moreover, we found that apelin expression was inhibited by TGF-β1 stimulation in MRC-5 fibroblasts, suggesting that TGF-β1 signaling activation in silicosis might be partly responsible for decreased apelin expression. In addition, we proposed that apelin inhibited TGF-β-SAMD2/3 pathway in fibroblasts, leading to the alleviation of silica-induced pulmonary fibrosis.

Since the discovery of the apelin/APJ interaction, numerous investigations have emerged highlighting its new roles in the regulation of different homeostatic processes and diseases. Apelin is widely distributed in pulmonary vascular endothelial cells, alveolar epithelial cells, bronchial epithelial cells 44, and fibroblasts 24. Accumulating reports had showed that apelin acts as an important protective modulator of lung diseases 23, 45. Pulmonary apelin levels are closely associated with respiratory diseases. The apelin levels in plasma and lung tissues were controversial. It reduced in patients with pulmonary hypertension 46 and chronic obstructive pulmonary disease 22, but elevated in patients with acute respiratory distress syndrome 20. A decrease in apelin levels was also observed in bleomycin-induced fibrotic mice 47. Although the link of apelin to several pulmonary diseases has been investigated, the association between apelin and silicosis is yet elusive 48. Therefore, one of the significant findings in this work is that we elucidated the association between apelin and silicosis. We found that the serum level and pulmonary expression of apelin reduced dramatically in both silicosis patients and silica-induced fibrotic mice, which indicates that decreased apelin levels might be associated with pulmonary fibrosis in silicosis. Moreover, it is noteworthy that silicosis patients with lower serum apelin levels exhibited impaired pulmonary function compared with the patients with higher serum apelin levels, suggesting that apelin might have a protective role in the pathogenesis of silicosis.

To date, although pirfenidone and nintedanib have been approved by the U.S. Food and Drug Administration (FDA) for idiopathic pulmonary fibrosis, no specific drug has been approved for silicosis in the United States and Europe yet 4. Current therapeutics are only able to delay the progression of silicosis or reduce complications. Therefore, the development of novel therapeutic approaches against pulmonary fibrosis is mandatory. A wide range of research demonstrated the anti-fibrotic effects of apelin on various organ fibrosis, including skin fibrosis 24, cardiac fibrosis 25, and renal fibrosis 27, 28. Referring to pulmonary fibrosis, several studies affirmed that apelin participated in the process of melatonin alleviating bleomycin-induced pulmonary injury and evodiamine alleviating lipopolysaccharide-induced pulmonary inflammation and fibrosis 47, 49. Apelin can also directly alleviate lipopolysaccharide-induced pulmonary fibrosis in mice by promoting angiotensin-converting enzyme 2 50 or suppressing TGF-β1 signaling 51. However, to date, most research focused on the protective effect of apelin on fibrosis by administrating apelin before or immediately after the stimulation 50, 51. To the best of our knowledge, this is the first study to show that the administration of apelin at the late stage could exhibit reversing pulmonary fibrosis induced by silica aside from its protective effect. When given at the early stage (day 3), apelin prevented histological damage and ECM accumulation in silica-induced fibrotic mice. Importantly, apelin also significantly attenuated the pathological changes and collagen accumulation even with a late treatment from day 15, when the fibrosis had already been established. Thus, our study provides overwhelming evidence for the use of apelin in the prevention and treatment of silica-induced pulmonary fibrosis.

Although apelin exerts effective preventing and alleviating effects against silica-induced pulmonary fibrosis, its underlying mechanism is unclear. Myofibroblasts are considered the primary effector cells and the source of ECM in the development of silicosis fibrosis. Although several types of cells can differentiate into myofibroblasts, FMT is considered of great importance 52, 53. During pulmonary fibrosis, fibroblasts differentiate into α-SMA-positive myofibroblasts, producing excessive ECM and causing pulmonary fibrosis 54. Preventing fibroblast activation or promoting myofibroblast apoptosis is necessary to resolute fibrosis 55. In this study, we used a macrophages-fibroblasts co-culture model. We found that upon the SiO_2_ stimulation, THP1-derived macrophages secreted TGF-β1 and activated MRC-5 fibroblasts. Our further study revealed that α-SMA levels and ECM deposition were significantly suppressed in TGF-β1-induced fibroblasts after being treated with apelin. Similar results were also observed in previous studies. Apelin may suppress cardiac fibroblast activation and profibrotic activity via sphingosine kinase 1-dependent mechanism 56. The administration of apelin and MM07 (a synthetic biased agonist of APJ) can also inhibit fibrosis-related gene expression in systemic sclerosis fibroblasts 24. We also noticed that apelin was reduced in TGF-β1-treated MRC-5 fibroblasts. This change may attenuate the inhibitory effect of apelin on TGF-β1 signaling and collagen production, resulting in the promotion of pulmonary fibrosis.

TGF-β/SMAD signaling is one of the classical pathways in driving the development and progression of tissue fibrosis 57. After binding to TGF-β receptor 2 (TGFR2), TGF-β1 recruits and activates the TGFR1. Then, the active TGFR1 phosphorylates SMAD2 and SMAD3, which form the SMAD complex with SMAD4. The complex translocates to the nucleus and triggers target gene transcription like *SNAI1* and *SNAI2,* leading to the consequently continuous expression of ECM proteins, such as a-SMA, fibronectin, and collagen I 8, 58. SMAD2 and SMAD3, the major downstream mediators, play a key role in the activation of fibroblasts and the pathogenesis of fibrosis 59. Literature demonstrated that the down-regulation of SMAD2/3 phosphorylation could prevent renal fibrosis 60. However, the overexpression of SMAD3 can reverse the inhibition effect of miR-497-5p on TGF-β1 induced-lung fibroblast activation 61. Likewise, we also observed that apelin suppresses the activation, migration, and ECM production of MRC-5 fibroblasts via TGF-β/SMAD signaling pathway. On the contrary, in the SMAD2/3-overexpressed fibroblasts, the fibrosis-inhibitory effect of apelin significantly declined. Several studies also found that the TGF-β/SMAD signaling might be the molecular mechanism of the inhibitory effects of apelin on organ fibrosis. Liu et al. suggested that the protective effect of apelin-13 on LPS-induced endothelial-to-mesenchymal transition was partially mediated by suppressing TGF-β/SMAD signaling pathway 51. Wang et al. found that apelin could block TGF-β/SMAD signaling in HK-2 cells and a unilateral ureteral obstruction induced renal fibrosis mouse model 42. In summary, our results suggested that inhibitory regulation by apelin may be mediated by inhibition of phosphorylation of SMAD2/3 and transcription factors *SNAI1* and *SNAI2 in vitro.* However, the precise mechanisms by which apelin inhibits TGF-β1/SMAD signaling are unknown, and further work is warranted to delineate the exact underlying mechanisms.

## Conclusion

In summary, we demonstrated that the lung expression and serum levels of apelin in silicosis patients and silicotic mice were significantly declined and associated with the severity. We also provided the first experimental evidence that apelin could prevent and reverse silica-induced fibrosis by suppressing the activation of pulmonary fibroblasts via inhibiting TGF-β1 pathway. This finding may provide new insight into the treatment of silicosis and other pulmonary fibrosis.

## Supplementary Material

Supplementary figures and tables.

## Figures and Tables

**Figure 8:**
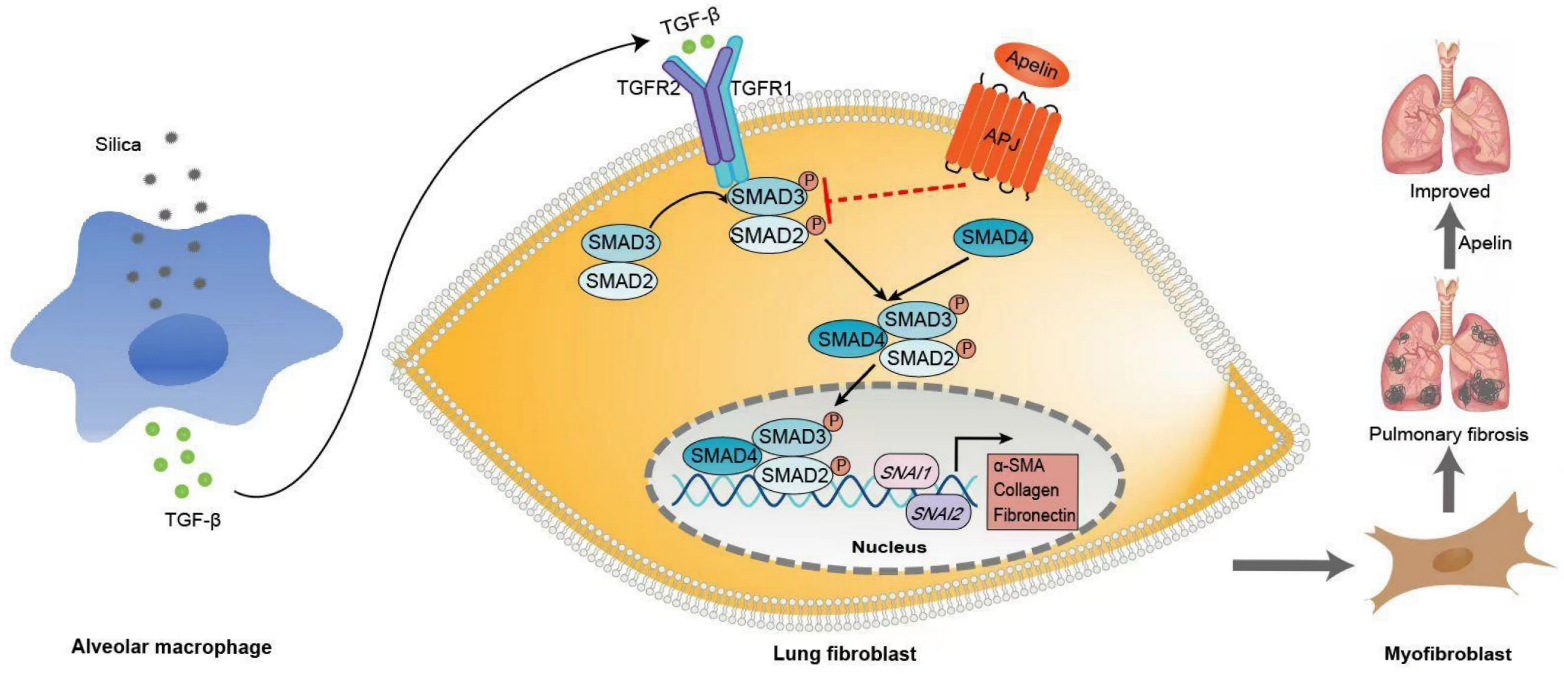
** Schematic representation of the mechanism by which apelin alleviates the progression of silica-induced pulmonary fibrosis.** During silica-induced pulmonary fibrosis, alveoli macrophages are activated by inhaled silica particles and release TGF-β. After binding with TGF-β, the TGF-β receptor is activated, resulting in the phosphorylation of SMAD2 and SMAD3. The phosphorylated SMAD2 and SMAD3 form the SMAD complex with SMAD4 and translocate to the nucleus to trigger target gene transcription, including transcription factors *SNAI1* and *SNAI2* and consequently the continuous expression of ECM proteins, such as fibronectin and collagen I. Apelin binding to and activating APJ inhibits the phosphorylation of SMAD2/3 and the expression of *SNAI1* and *SNAI2*, suppresses lung fibroblast activation and ECM production triggered by TGF-β, thus alleviates pulmonary fibrosis.

## Abbreviations

APJ: apelin receptor
TGF-β: transforming growth factor beta
TNF-ɑ: tumor necrosis factor-ɑ
FMT: fibroblast-to-myofibroblast transition
ECM: extracellular matrix
DLCO: diffusing capacity of the lungs for carbon monoxide
TLC: total lung capacity
FVC: forced vital capacity
FEV1: forced expiratory volume in one second
FEF50%: forced expiratory flow at 50% of the pulmonary volume
H&E: hematoxylin-eosin
IHC: immunohistochemistry
RT: room temperature
α-SMA: alpha-smooth muscle actin
CCK8: cell counting kit-8
IF: immunofluorescence
DAPI: 4',6-Diamidino-2-phenylindole
ELISA: enzyme-linked immunosorbent assay
RT-qPCR: real-time quantitative PCR
SMAD2: SMAD family member 2
SMAD3: SMAD family member 3
ANOVA: one-way analysis of variance
Collagen I: collagen type I
TGFR1: TGF-β receptor 1
p-SMAD2/3: phosphorylated SMAD2/3
*SNAI1*: snail family transcriptional repressor 1
*SNAI2*: snail family transcriptional repressor 2
TGFR2: TGF-β receptor 2
