# Supplementary material for: Apelin Prevents and Alleviates Crystalline Silica-induced Pulmonary Fibrosis via Inhibiting Transforming Growth Factor Beta 1-triggered Fibroblast Activation
Source: Int J Biol Sci. 2023 Jul 31;19(13):4004–19. doi: 10.7150/ijbs.81436 (PMC10496498; doi:10.7150/ijbs.81436)
Supplement: Supplementary file 1 — Supplementary figures and tables. [file ijbsv19p4004s1.pdf]

Supplementary Materials

Apelin Prevents and Alleviates Crystalline Silica-induced Pulmonary Fibrosis via Inhibiting Transforming Growth Factor Beta 1-triggered Fibroblast Activation

Jianling Shen[1,2]#, Jiayin Feng[1,2], Zhijia Wu[1,3], Yushi Ou[1,2], Qing Zhang[1,4], Qiying Nong[1], Qifeng Wu[1], Cong Li[1], Xiaohui Tan[5], Meng Ye[6], Zhongxiang Gao[1], Ying Zhang[1], Weihui Liang[1], Lihua Xia[1], Yiru Qin[1]*, Yongshun Huang[1,2,3]*, Na Zhao[1,2,3]*, Shijie Hu[1]

1. Guangdong Provincial Key Laboratory of Occupational Disease Prevention and Treatment, Guangdong Province Hospital for Occupational Disease Prevention and Treatment, Guangzhou, China.
2. School of Public Health, Sun Yat-Sen University, Guangzhou, China.
3. School of Public Health, Southern Medical University, Guangzhou, China.
4. Pudong New Area Center for Disease Control and Prevention, Shanghai, China.
5. Guangzhou Key Laboratory of Forensic Multi-Omics for Precision Identification, School of Forensic Medicine, Southern Medical University, Guangzhou, China.
6. National Institute for Occupational Health and Poison Control, Chinese Center for Disease Control and Prevention, Beijing, China.

#Jianling Shen was First Author

*Corresponding authors: **Yiru Qin**, Guangdong Provincial Key Laboratory of Occupational Disease Prevention and Treatment, Guangdong Province Hospital for Occupational Disease Prevention and Treatment, Guangzhou, Guangdong 510300, China. Tel: 86-20-34066482; Email: yiru@mail.usf.edu. **Yongshun Huang**, Guangdong Provincial Key Laboratory of Occupational Disease Prevention and Treatment, Guangdong Province Hospital for Occupational Disease Prevention and Treatment, Guangzhou, Guangdong 510300, China. Tel: 86-20-34063101; Email: gdpcc2006@126.com. **Na Zhao**, Guangdong Provincial Key Laboratory of Occupational Disease Prevention and Treatment, Guangdong Province Hospital for Occupational Disease Prevention and Treatment, Guangzhou, Guangdong 510300, China. Tel: 86-20-34063251; Email: zhaonabmu@126.com.

Supplementary Figures

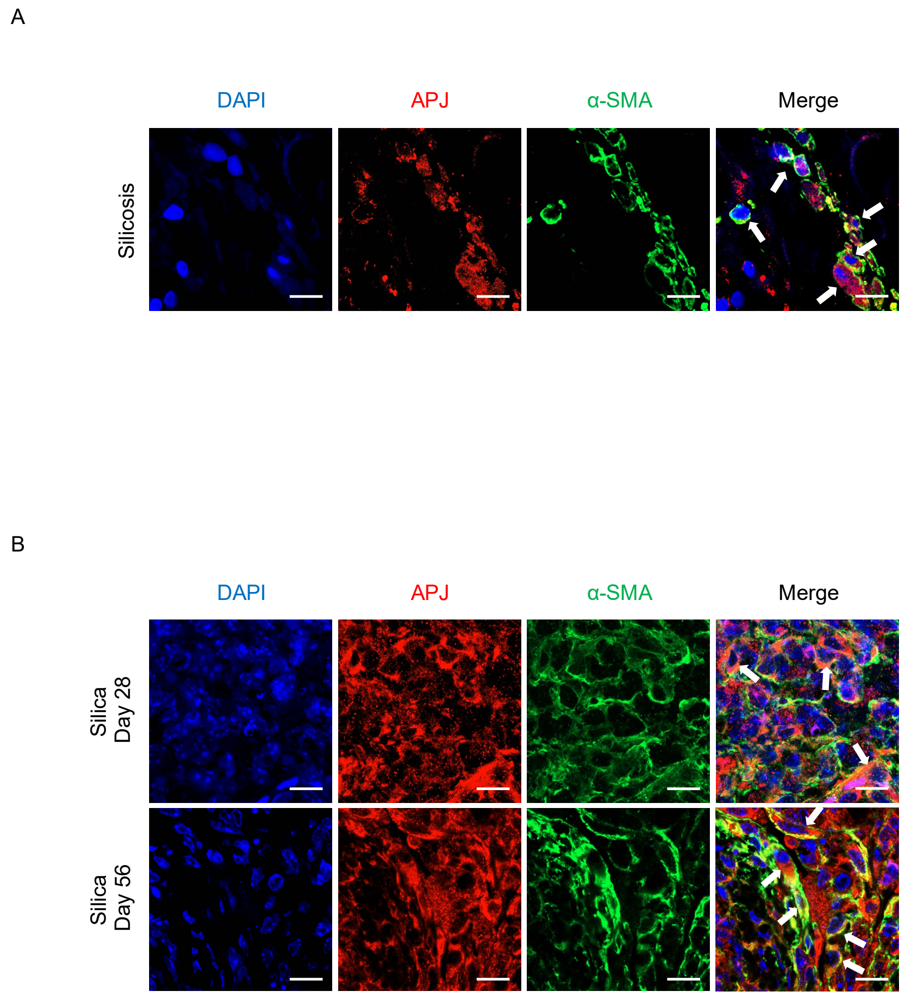

Figure S1. APJ expression in activated fibroblasts of silicosis patients and silicotic mice. (A) Confocal images of APJ and α-SMA double-immunostaining in lung tissues from silicosis patients. **(B)** Confocal images of APJ and α-SMA double-immunostaining in lung tissues from silica- treated mice. Cells were counterstained with DAPI to visualize nuclei. The white arrows show the colocalization of APJ and α-SMA protein in activated fibroblasts. Scale bar: 4 µm.

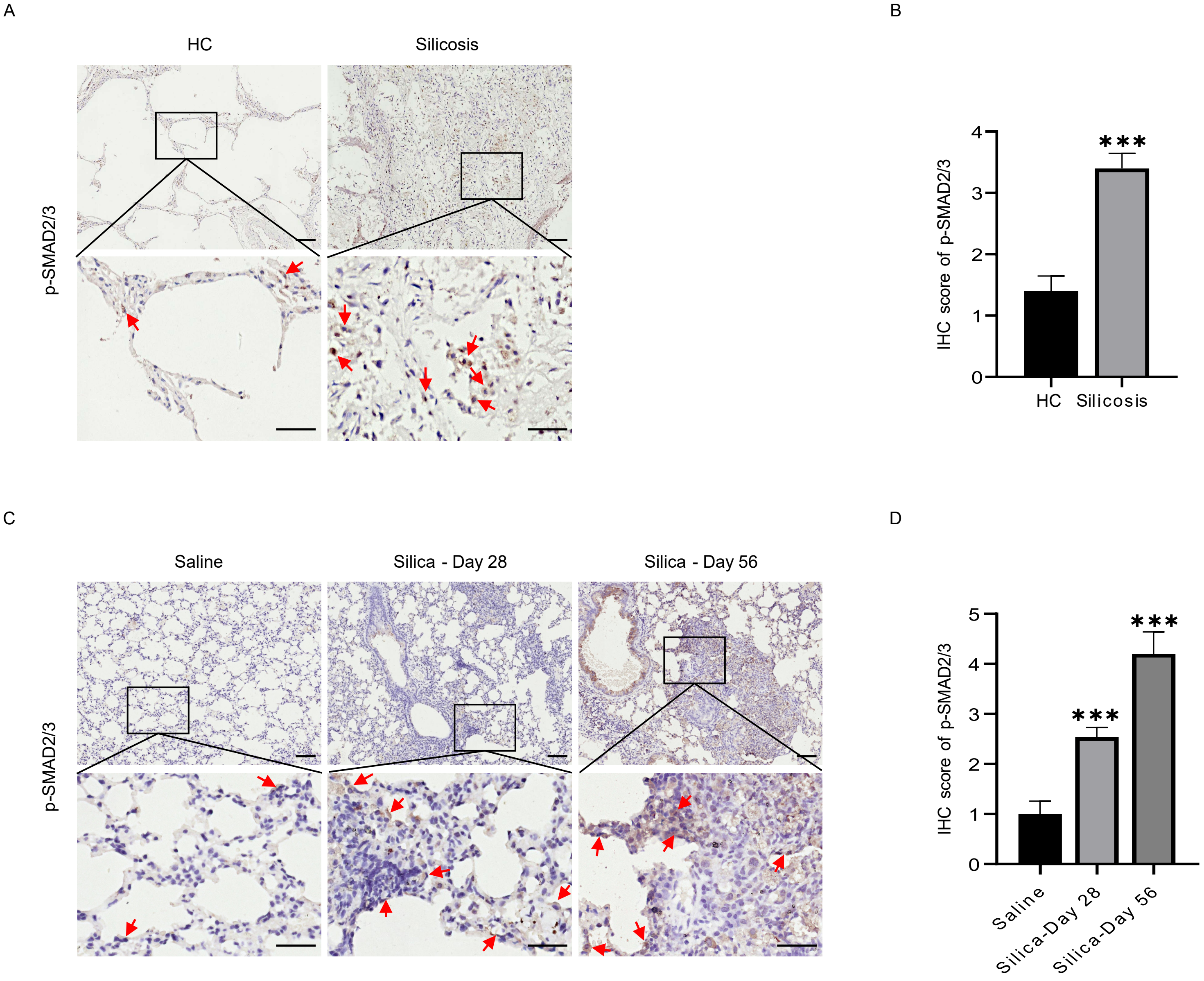

Figure S2. p-SMAD2/3 expression in silicosis patients and silicotic mice. (A) Representative images of p-SMAD2/3 immunostaining in lung tissues from healthy controls and silicosis patients. The boxed regions are shown at higher magnification in the down panels. The red arrows show positive cells. Scale bar: 25 μm. **(B)** The immunohistochemical scores of p-SMAD2/3 in healthy controls (n=6) and silicosis patients (n=5). **(C)** Representative images of p-SMAD2/3 immunostaining in lung tissues from saline- and silica- treated mice. **(D)** The immunohistochemical scores of p-SMAD2/3 in saline- and silica- treated mice (n=5). Data are presented as means ± SEM for at least triplicate experiments. $P > 0.05$ is considered not significant, and $^{***}P < 0.001$.

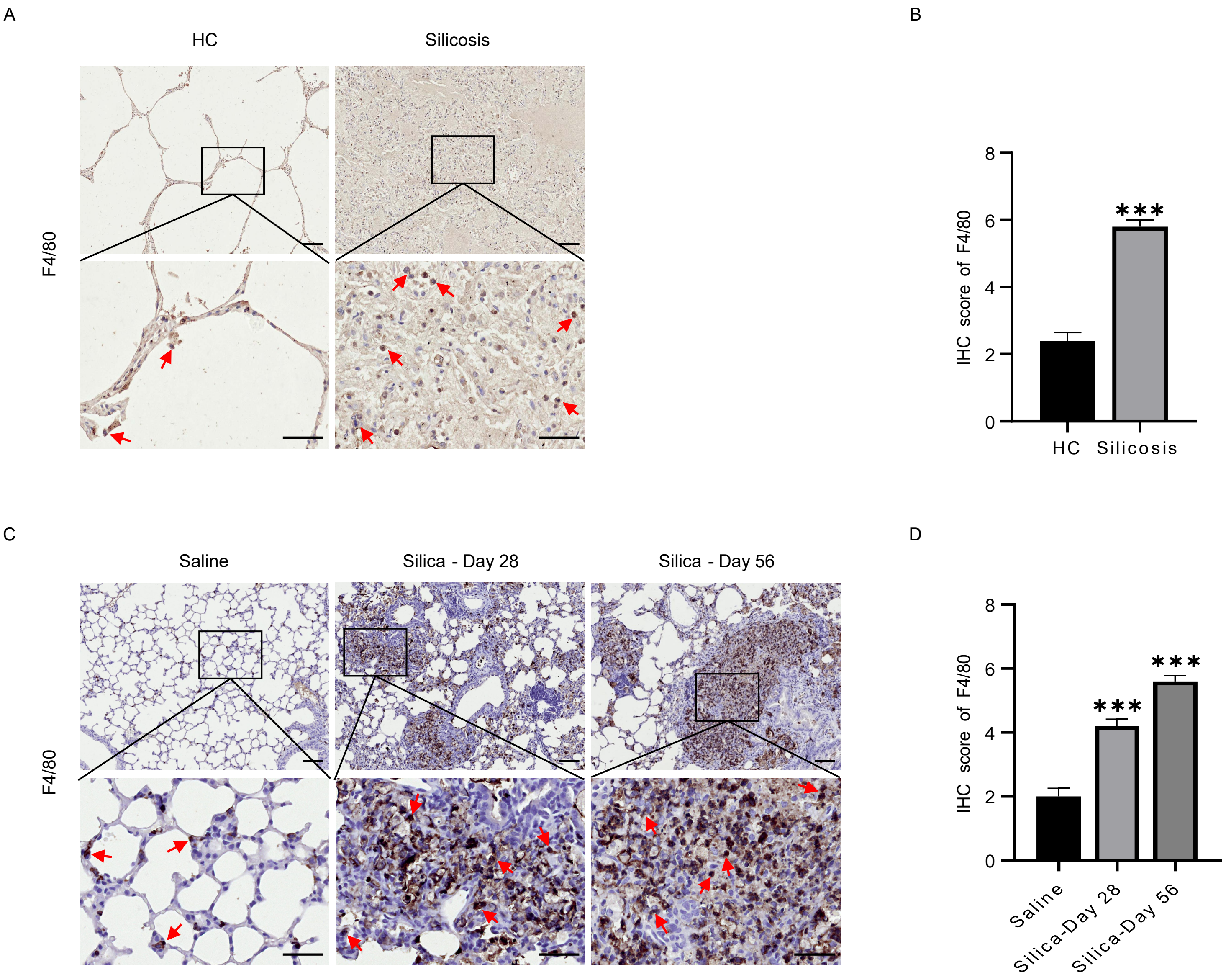

Figure S3. F4/80 expression in silicosis patients and silicotic mice. (A) Representative images of F4/80 immunostaining in lung tissues from healthy controls and silicosis patients. The boxed regions are shown at higher magnification in the down panels. The red arrows show positive cells. Scale bar: 25 μm. **(B)** The immunohistochemical scores of F4/80 in healthy controls (n=6) and silicosis patients (n=5). **(C)** Representative images of F4/80 immunostaining in lung tissues from saline- and silica- treated mice. **(D)** The immunohistochemical scores of F4/80 in saline- and silica- treated mice (n=5). Data are presented as means ± SEM for at least triplicate experiments. $P > 0.05$ is considered not significant, and $^{***}P < 0.001$.

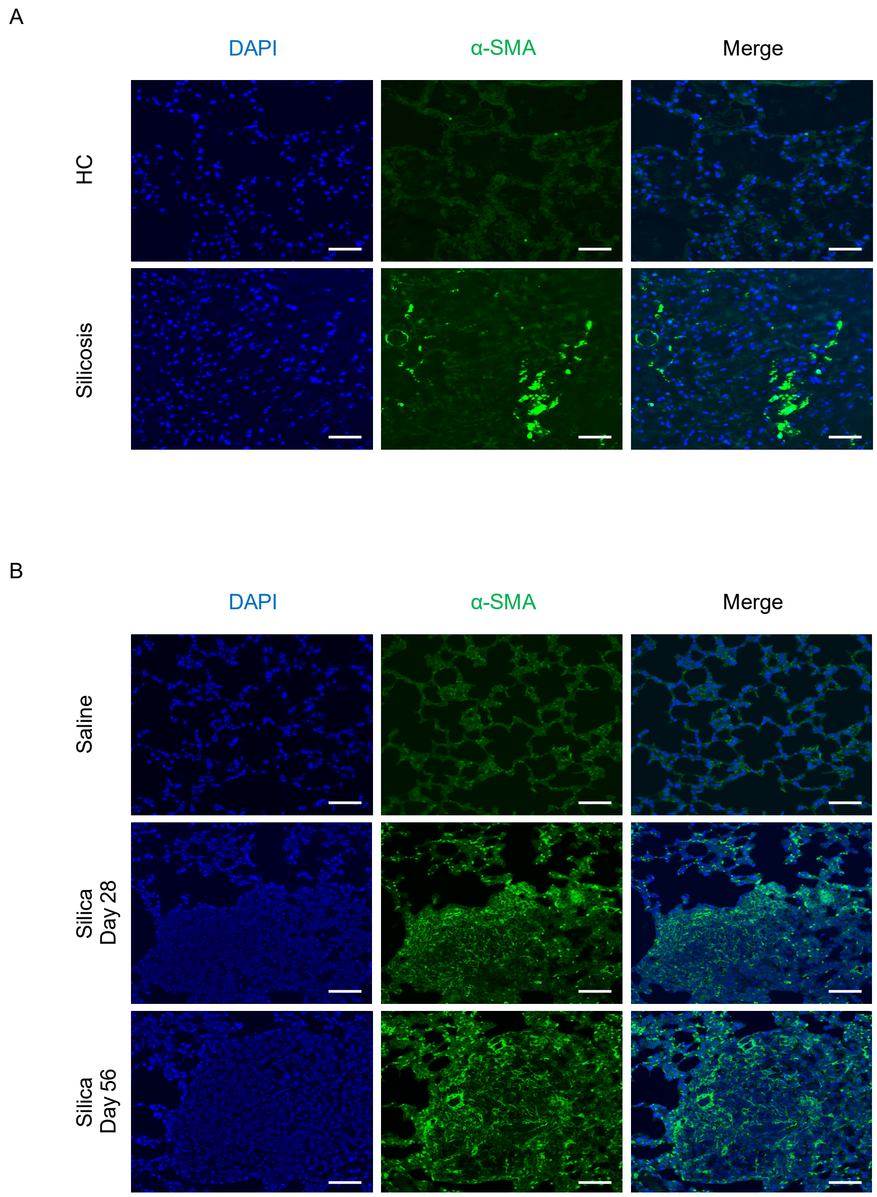

Figure S4. α-SMA expression in silicosis patients and silicotic mice. (A) Representative images of α-SMA immunostaining in lung tissues from healthy controls and silicosis patients. **(B)** Representative images of α-SMA immunostaining in lung tissues from saline- and silica-treated mice sacrificed on day 28 and 56. Cells were counterstained with DAPI to visualize nuclei. Scale bar: 25 μm.

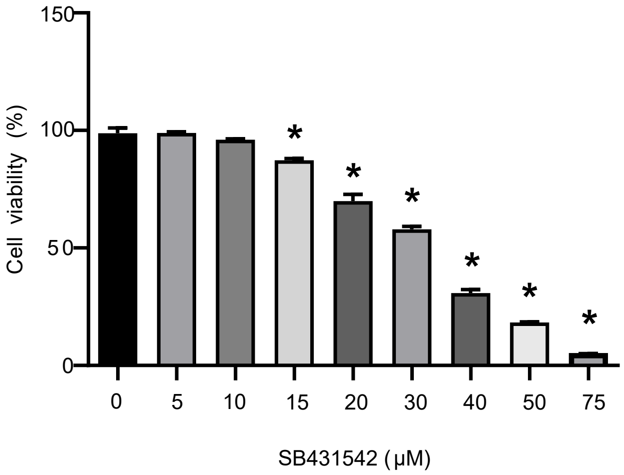

Figure S5. Cytotoxicity of SB431542 in MRC-5 cells. Cell viability of MRC-5 cells that had been treated with different doses of SB431542 for 48 hours assessed by CCK8 assay. Data are presented as means ± SEM for at least triplicate experiments. *P* > 0.05 is considered not significant, **P* < 0.05.

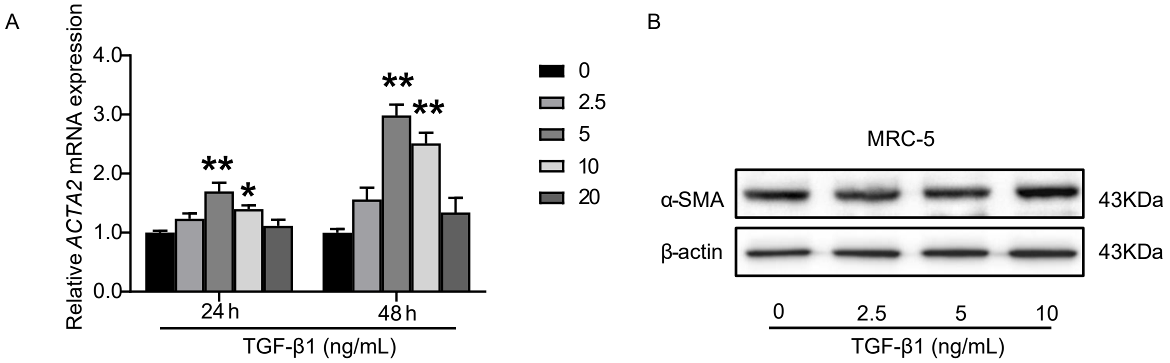

Figure S6. TGF-β1 induces the expression of α-SMA in MRC-5 fibroblasts. (A) The mRNA expression of *ACTA2* in MRC-5 cells after treatment with different doses of TGF-β1 for 24 hours and 48 hours. **(B)** Western blotting analysis of α-SMA expression in MRC-5 cells after treatment with different doses of TGF-β1 for 48 hours. β-actin was used as a loading control. Data are presented as means ± SEM for at least triplicate experiments. $P > 0.05$ is considered not significant, $*P < 0.05$, and $**P < 0.01$.

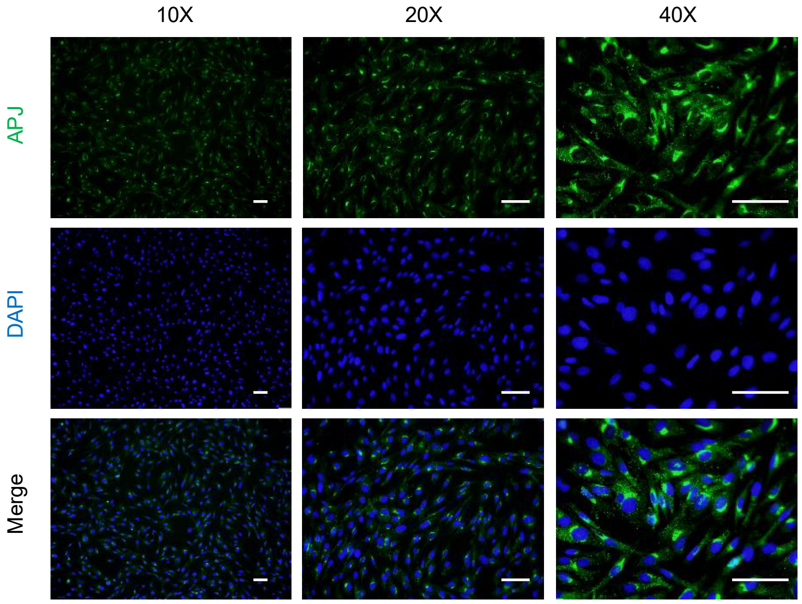

Figure S7. **APJ expression in MRC-5 fibroblasts.** Representative images of APJ immunostaining in MRC-5 cells. Cells were counterstained with DAPI to visualize nuclei. Scale bar: 25 µm.

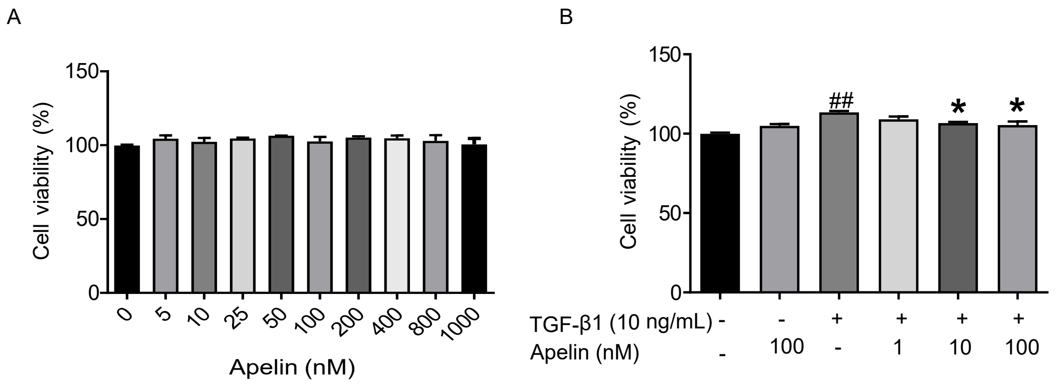

Figure S8. Cytotoxicity of apelin in MRC-5 fibroblasts. (A) Cell viability of MRC-5 cells that had been treated with different doses of apelin for 48 hours assessed by CCK8 assay. **(B)** Cell viability of MRC-5 cells that had been treated with or without TGF-β1 and apelin for 48 hours assessed by CCK8 assay. Data are presented as means ± SEM for at least triplicate experiments. $P > 0.05$ is considered not significant, $^{##}P < 0.01$ *vs.* vehicle group, and $^{*}P < 0.05$ *vs.* TGF-β1 group.

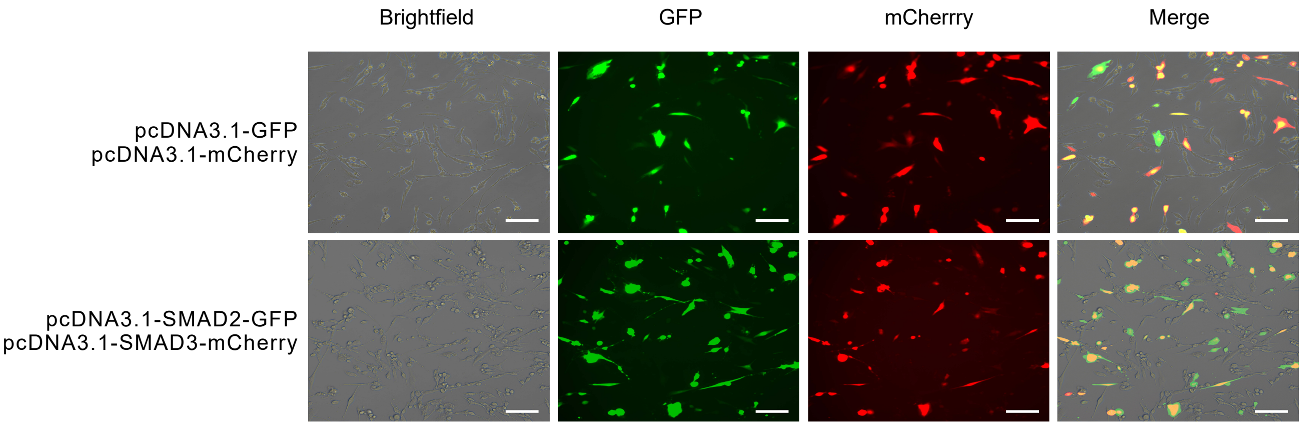

Figure S9. Transfection efficiency of MRC-5 fibroblasts. Brightfield, GFP fluorescence, mCherry fluorescence, and their merged images of MRC-5 cells after co-transfection with pcDNA3.1-SMAD2-GFP and pcDNA3.1-SMAD3-mCherry plasmid or pcDNA3.1-GFP and pcDNA3.1-mCherry plasmid for 24 hours. Scale bar: 50 µm.

Supplementary Tables

Table S1. Antibodies used in this study

Antibody	Application	Company
APJ	1:1000 for cell IF; 1:1000 for IHC; 1:1000 for WB; 1:400 for tissue IF	Proteintech, USA
α-SMA	1:200 for cell IF; 1:1000 for tissue IF; 1:1000 for WB	Abcam, UK
Apelin	1:100 for IHC; 1:1000 for WB	Proteintech, USA
Fibronectin	1:1000 for WB	Abcam, UK
Collagen I	1:1000 for WB	Abcam, UK
SMAD2/3	1:1000 for WB	Cell Signaling, USA
p-SMAD2	1:1000 for WB	Cell Signaling, USA
p-SMAD3	1:1000 for WB	Cell Signaling, USA
p-SMAD2/3	1:200 for IHC	Affinity, China
F4/80	1:500 for IHC	Cell Signaling, USA
F4/80	1:1000 for IHC	Proteintech, USA
β-actin	1:1000 for WB	Abcam, UK
Goat Anti-Rabbit IgG H&L (Alexa Fluor® 568)	1:200 for IF	Abcam, UK
Goat Anti-Mouse IgG H&L (Alexa Fluor 488)	1:200 for IF	Abcam, UK

Table S2. Sequences information used in this study

Name	Sequence
β-actin Forward	GGCACCCAGCACAATGAAG
β-actin Reverse	CCGATCCACACGGAGTACTTG
ACTA2 Forward	AAAAGACAGCTACGTGGGTGA
ACTA2 Reverse	GCCATGTTCTATCGGGTACTTC
Fibronectin Forward	CACTTACCGAGTGGGTGACACTT
Fibronectin Reverse	GCAGGTACAGTCCCAGATCATG
COL1A1 Forward	CGGAGGAGAGTCAGGAAGG
COL1A1 Reverse	CACAAGGAACAGAACAGAACA
APLN Forward	GTCTCCTCCATAGATTGGTCTGC
APLN Reverse	GGAATCATCCAAACTACAGCCAG
APLNR Forward	CTCTGGACCGTGTTTCGGAG
APLNR Reverse	GGTACGTGTAGGTAGCCCACA
SNAI1 Forward	CCCCAATCGGAAGCCTAACT
SNAI1 Reverse	CGTAGGGCTGCTGGAAGGTA
SNAI2 Forward	CCATTCCACGCCCAGCTA
SNAI2 Reverse	CTCACTCGCCCCAAAGATGA
Apln Forward	GTCTCCTCCATAGATTGGTCTGC
Apln Reverse	GGAATCATCCAAACTACAGCCAG
Aplnr Forward	CTCTGGACCGTGTTTCGGAG
Aplnr Reverse	GGTACGTGTAGGTAGCCCACA
